# Gestational alcohol exposure disrupts cognitive function and striatal circuits in adult offspring

**DOI:** 10.1038/s41467-020-16385-4

**Published:** 2020-05-22

**Authors:** Verginia C. Cuzon Carlson, Christina M. Gremel, David M. Lovinger

**Affiliations:** 1 Laboratory for Integrative Neuroscience, National Institute on Alcohol Abuse and Alcoholism, National Institutes of Health, 5625 Fishers Lane, Bethesda, MD 20892 USA; 2 Division of Neuroscience, Oregon National Primate Research Center, Oregon Health & Science University, 505 NW 185th Ave, Beaverton, OR 97006 USA; 3 Department of Psychology and The Neurosciences Graduate Program, University of California San Diego, 9500 Gilman Drive, #0109, La Jolla, CA 92093-0109 USA

**Keywords:** Addiction, Operant learning

## Abstract

Fetal alcohol exposure (FAE) is the leading preventable developmental cause of cognitive dysfunction. Even in the absence of binge drinking, alcohol consumption during pregnancy can leave offspring deficient. However, the mechanisms underlying these deficiencies are unknown. Using a mouse model of gestational ethanol exposure (GEE), we show increased instrumental lever-pressing and disruption of efficient habitual actions in adults, indicative of disrupted cognitive function. In vivo electrophysiology reveals disrupted action encoding in dorsolateral striatum (DLS) associated with altered habit learning. GEE mice exhibit decreased GABAergic transmission onto DLS projection neurons, including inputs from parvalbumin interneurons, and increased endocannabinoid tone. Chemogenetic activation of DLS parvalbumin interneurons reduces the elevated lever pressing of GEE mice. Pharmacologically increasing endocannabinoid tone mimics GEE effects on cognition and synaptic transmission. These findings show GEE induces long-lasting deficits in cognitive function that may contribute to human FAE, and identify potential mechanisms for future therapeutic targeting.

## Abstract (web summary)

Alcohol is the leading cause of preventable birth defects in the US, collectively referred to as Fetal Alcohol Spectrum Disorder. Here, the authors show that fetal alcohol exposure induces lasting neurophysiological changes in dorsal striatum that contribute to less efficient decision making.

## Introduction

Although most women reduce drinking during pregnancy, fetal alcohol exposure (FAE) is the leading preventable developmental cause of cognitive dysfunction worldwide, constituting a major public health issue with severe economic cost^1^. While the consequences of severe FAE are readily observable^2,3^, moderate alcohol consumption during pregnancy may result in fetal alcohol spectrum disorder (FASD), in part characterized by cognitive deficits^1^, including slower processing speed and greater cognitive effort^4^. Structural and functional changes in cortico-basal ganglia circuits controlling cognitive function likely contribute to these deficiencies^5–8^. However, our understanding of the specific cellular and learning disruptions is surprisingly limited.

Cognitive function involves decision-making processes, where the use of efficient strategies must be balanced with the need to reevaluate and adjust under changing circumstances. This balance in decision-making is often executed through a combination of action strategies that rely on efficient habitual strategies versus more cognitively demanding goal-directed strategies^7^. The learning of actions and their control by decision-making strategies depends upon the main input structure of the basal ganglia, the dorsal striatum (DS). Interestingly, there is regional segregation within the DS with respect to learned actions and decision-making strategies. Well-learned, generalized actions, and use of habitual strategies, involve the dorsolateral striatum (DLS)^7–15^ while action discrimination, and use of goal-directed strategies depend more upon the dorsomedial striatum (DMS)^10,14,16–18^. Learned actions controlled by habitual and goal-directed strategies are fundamental for efficient but flexible cognitive function^7^, which is often perturbed in FASD^1^.

To identify molecular mechanisms underlying disrupted cognitive function, we examined the lasting effects of gestational ethanol (EtOH) exposure (GEE) on DS function in adult mice. We found that GEE increased lever-pressing and biased against habitual action strategies in an instrumental task tested in adulthood, mirrored by disrupted action encoding in the DLS. Furthermore, these dysfunctional cognitive phenotypes were accompanied by decreased GABAergic transmission onto DLS medium spiny neurons (MSNs), concomitant with increased endocannabinoid (EC) tone. In particular, we identified a decrease in synaptic transmission from parvalbumin-expressing interneurons (PVs) to MSNs (PV-MSN), due in part to altered EC control. Chemogenetic reversal of MSN disinhibition and pharmacological manipulation of EC tone confirmed their involvement in proper control of action rates and decision-making strategies, respectively. Increases in EC tone mimicked, while decreases partially rescued, GEE effects on DLS GABAergic transmission. We show that in the absence of the most severe FAE symptoms, moderate GEE induces lasting cognitive dysfunction, and we identified novel mechanisms in DLS underlying this disastrous complex phenotype.

## Results

### Mouse model of gestational alcohol exposure

**Fig. 1:**
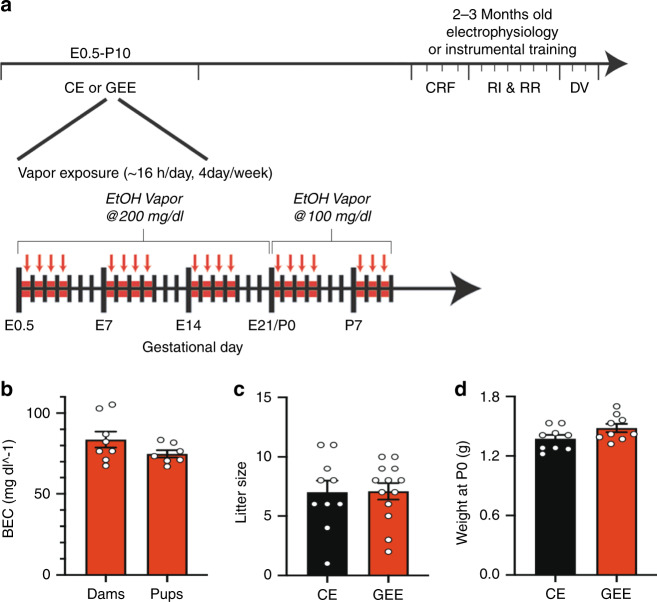
Gestational ethanol exposure (GEE) paradigm. **a** Schematic diagram of the GEE paradigm. Mice were exposed to ethanol or air (CE) for 16 hours/day for 4 days/week from embryonic day (E)0.5 to postnatal day (P)10. The ethanol vapor concentration averaged 200 mg/dl from E0.5-E21. After birth the ethanol concentration was lowered to average 100 mg/dl. At 2–3 months, mice were either used for ex vivo electrophysiology or instrumental training (continuous reinforcement schedules (CRF), training under random interval (RI) and random ratio (RR) schedules, and outcome devaluation (DV) tests). **b** GEE elicited similar blood ethanol concentrations (BEC) in dams (from E0.5-E21.5) and pups (from P0-P10). However, GEE did not alter (**c**) litter size or (**d**) body weight of offspring at birth. Black bars = CE mice; red bars = GEE mice. Error bars equal ± SEM.

We modified a mouse vapor inhalation model^19–26^ to mimic alcohol exposure during a period similar to the three trimesters of human gestation. Upon detection of a seminal plug (embryonic day 0.5), pregnant mice were exposed to EtOH vapor (200 mg/dl) through day of birth (postnatal day (P) 0) (Fig. 1a), yielding blood EtOH concentrations (BECs) averaging 83.7 ± 4.9 mg/dl in the pregnant dam (Fig. 1b). The average litter size and birth weight were unaffected by GEE (unpaired *t*-test; litter size: *p* = 0.95; body weight at P0: *p* = 0.08) (Fig. 1c, d). To mimic exposure during the third trimester of human gestation that occurs during the early postnatal period in rodents^26,27^, the same dams and litters were exposed to a reduced level of EtOH vapor (100 mg/dl) from P0 to P10 (Fig. 1a) resulting in moderate BECs in pups (74.9 ± 2.3 mg/dl) that was similar to the BECs measured in pregnant dams (unpaired *t*-test compared to dams during pregnancy, *p* = 0.15) (Fig. 1b). During this postpartum period of reduced ethanol exposure dams did not have a measurable BEC. Normal maternal care was observed by proper nesting behavior, presence of a milk spot, and similar body weights from P0–P10 (Supplementary Fig. 11d) (“Methods”) between GEE and control mice exposed to air vapor (CE). We did not observe facial dysmorphology or gross physical deformities typical of individuals with FAS or mice after more severe EtOH exposures^2,28,29^.

### GEE alters cognitive function examined in adulthood

**Fig. 2:**
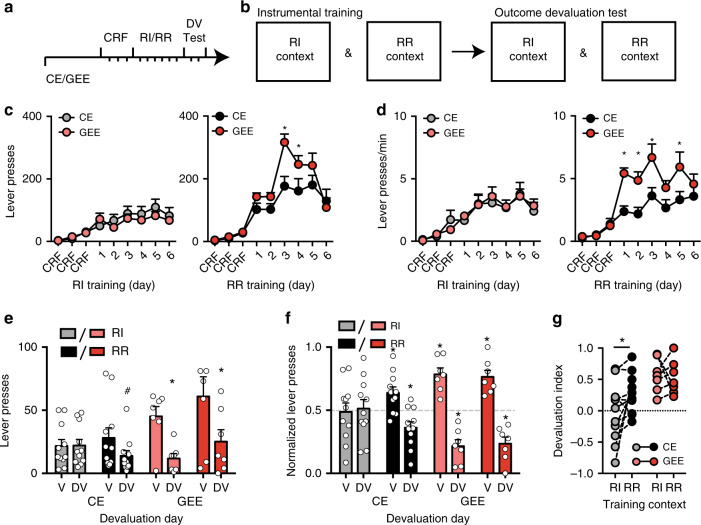
Gestational ethanol exposure (GEE) disrupts cognitive function. **a** Schematic diagram of experimental time course. At 2–3 months of age a subset of mice was used for instrumental training (continuous reinforcement schedules (CRF), training under random interval (RI) and random ratio (RR) schedules, and outcome devaluation (DV) tests). **b** Schematic of instrumental training. Mice were trained each day in two separate operant chambers that were distinguished by contextual cues denoting either RI or RR schedules. Outcome devaluation testing occurred across two consecutive days (the valued day and devalued day), with testing occurring in each context. **c** Lever presses and **d** response rate during RI and RR schedule training for CE and GEE mice. **e** Lever presses and **f** normalized lever-pressing in RI and RR trained contexts during outcome devaluation testing in Valued (V) and Devalued (DV) states. **g** Devaluation index of individual mice tested under RI and RR contexts. Error bars equal ± SEM, Repeated measures ANOVA Bonferroni corrected *p < 0.05, ^#^*p* < 0.06.

To assess long-lasting effects of GEE on cognitive processes in adulthood (2–3 months of age), we used a within subject design of concurrent instrumental training under random ratio (RR: goal-directed)^30,31^ and random interval (RI: habit)^32^ schedules followed by outcome devaluation procedures, to examine operationally defined goal-directed or habitual decision-making strategies, respectively, controlling action execution^9,18,30,33,34^ (see “Methods”). We uncovered two main phenotypic differences in GEE mice: enhanced lever-presses during RR training (Fig. 2c, d) and strong devaluation in both contexts indicating a disruption in the use of habitual action strategies (Fig. 2e–g).

Enhanced response rate emerged in GEE mice during schedule training. All mice were able to lever press and increased lever-pressing across training (main effect Training day *F*s’ > 14.63, *p*s’ < 0.001). However, GEE mice lever-pressed more (Fig. 2c) and had a higher press rate (Fig. 2d) than CE mice during RR schedule training (RR schedule interaction: *F*s’ > 2.99, *p*s’ < 0.01; main effect of Group: *F*s’ > 4.70, *p*s < 0.05) (RI schedule: no interaction or main effect, *p*s’ > 0.05), without differences in head entries and rewards earned between treatment groups (Supplementary Fig. 1a, b). This was not observed during RI training, indicating that increased responding is specific to the RR schedule where response rate controls rate of rewards^35^. The heightened response rate across training does not appear to be explained by a GEE-induced generalized hyperactivity (Supplementary Fig. 1c, d). These findings suggest that although GEE mice learn to make self-initiated actions for food, they make actions at a higher rate during training.

Strikingly, GEE produced an alteration in decision-making strategy in mice. When we examined lever-presses following outcome devaluation (Methods), CE mice reduced lever-pressing in the RR context, but not in the RI context, while GEE mice reduced lever-pressing in both contexts (Fig. 2e) (repeated measures ANOVA (Treatment x Valuation state) interaction: F3, 32 = 5.19, *p* < 0.01) (Bonferroni corrected *p*s’ < 0.05) (main effect of Valuation state: F1, 16 = 9.12 *p* < 0.01, no main effect of Treatment *p* > 0.05). Although GEE mice showed heightened levels of responding in the valued state (V), in the devalued state (DV) they still reduced responding following outcome devaluation in both RI and RR training contexts (Bonferroni corrected *p*s < 0.05). There were no differences in head entries during training, outcome revaluation testing and consumption prior to testing (Supplementary Fig 1e, f).

To determine if the enhanced response rates contributed to the distribution of lever presses we examined how individual CE and GEE mice distributed their lever-pressing between V and DV (Fig. 2f) by normalizing lever-presses in each state to the total lever-pressing in that context. We found that CE mice differently distributed their lever-pressing only in the RR context (one-sample *t*-test against 0.5, *t*s’ > 2.94, *p*s’ < 0.02), but not in the RI context (*p*s’ > 0.05), suggesting a shift between using a habitual strategy in the RI context and a goal-directed strategy in the RR context. In contrast GEE mice made more of their lever presses in the V and less in the DV in both training contexts (one-sample *t*-test against 0.5 for RR and RI training contexts, *t*s’ > 5.13, *p*s’ < 0.01). The apparent lack of a shift between action strategies in GEE mice was clearly evident when we examined the magnitude of goal-directedness expressed by individual mice across contexts, as measured by a devaluation index (Fig. 2g) (Lever-presses (V − DV)/ Lever-presses (V + DV)). While the CE mice showed stronger goal-directed control in the RR than RI context (paired *t*-test (RR vs RI), *t* = 1.99, *p* < 0.05), GEE mice showed a similar magnitude of goal-directedness in RR and RI training contexts (paired *t*- test, *p* = 0.8). A two-way repeated measures ANOVA showed a main effect of group (F1, 16 = 0.65, *p* = 0.006), but no effect of schedule and interaction, indicating that GEE mice were overall more goal-directed. The lack of habitual control in GEE mice was still present after 6 additional days of RI training (Supplementary Fig. 1g), suggestive of a persistent phenotype. Further, this habitual behavior did not appear to be due to enhanced goal-directed learning, as GEE mice trained only on the habit-biasing RI schedule still showed strongly decreased lever pressing on the DV day (Supplementary Fig. 1h). These results suggest that (1) increased lever-pressing during learning and devaluation phenotypes are separable and (2) GEE induces developmental changes that result in a long-lasting inability to use efficient habitual action strategies.

### GEE alters action-encoding in DLS

**Fig. 3:**
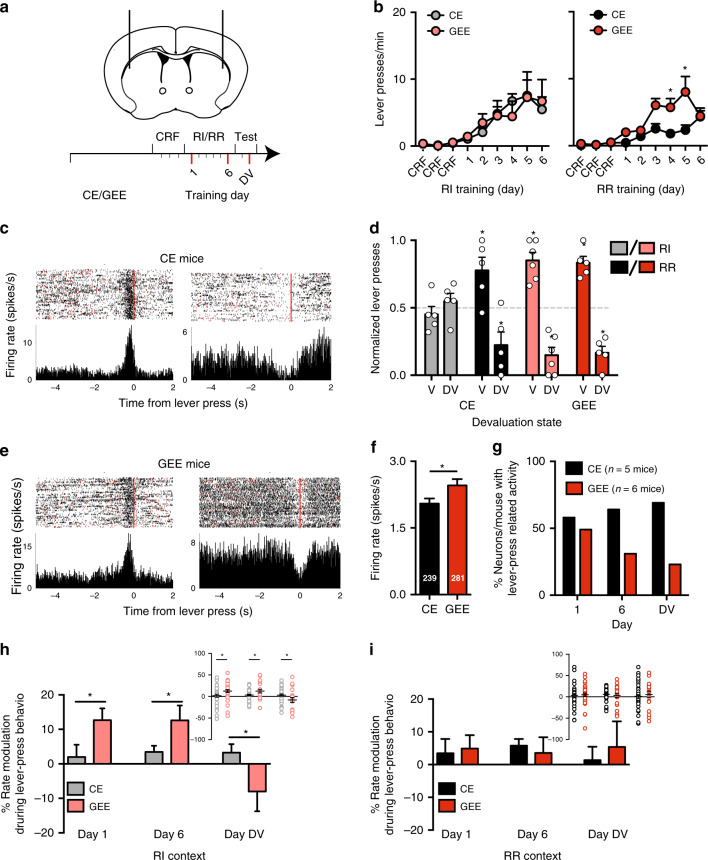
GEE disrupts in vivo habitual action encoding in DLS circuits. **a** Schematic diagram showing site of multi-electrode recording in the DLS (black) and DMS (gray), and experimental design. In vivo recordings were made on day 1 and 6 of schedule training and outcome devaluation (DV) testing (marked in red). (**b**) Response rate during RI and RR schedule training for CE and GEE mice that were implanted with multi-electrode arrays. **c**, **e** Representative raster plots (top-panel) and peri-event time histograms (bottom-panel) of putative DLS MSNs increasing (left) or decreasing (right) their activity around the lever-press (red line at time 0 (s)) in CE (**c**) and GEE (**e**) mice. Each row in the raster is neural activity +5 to −2 s around a lever press (time = 0 s, red line). Trials are sorted according to the order of lever-presses made across the session. The peri-event time histogram shows the average firing rate during lever-press related behavior. **d** Normalized lever-pressing in RI and RR trained contexts during outcome revaluation testing in Valued (V) and Devalued (DV) states in CE and GEE mice implanted with multi-electrode arrays. **f** Baseline firing rate of putative DLS MSNs that fired in both RI and RR training contexts in CE (*n* = 239) and GEE (*n* = 281) mice. **g** Proportion of putative DLS MSNs per animal that changed firing rate during lever-pressing under RI and RR schedules during training (day 1 and day 6) and following outcome devaluation. **h**, **i** The % rate net modulation during lever-press related behavior of firing rate in CE and GEE mice in **h** RI and **i** RR contexts. Inset shows percentage rate modulation distribution of individual units on Days 1, 6, and DV. Error bars equal ± SEM, * = *p* < 0.05.

Actions and decision-making strategies are controlled through circuits that include the DS. The GABAergic MSNs make up >90% of DS neurons, and constitute the sole output to downstream basal ganglia regions. In vivo physiology experiments show that firing of the same MSN encodes actions during both RI and RR schedule training as well as during performance of goal-directed and habitual actions during outcome devaluation testing^14^. We used chronic indwelling multi-electrode arrays to record the activity of putative DLS and DMS MSNs from CE (*n* = 5) or GEE (*n* = 6) mice (Supplementary Fig. 3a, b), during training and outcome devaluation testing (Methods) (Fig. 3a).

Mice chronically implanted with multi-electrode arrays increased the number and rate of lever-pressing across training (main effect of Training day: *F*s’8, 72 > 8.35, *p*s’ < 0.001) (Fig. 3b) (Supplementary Fig. 2a). Once again, GEE mice showed an enhanced response rate in the RR context (repeated measures ANOVA (Training Day × Treatment) interaction; F8, 72 = 3.13, *p* < 0.01) (Bonferroni corrected *p*s’ < 0.05)(Fig. 3b), while displaying similar head entries and rewards earned (Supplementary Fig. 2b-c). During subsequent outcome devaluation testing, on the DV day CE mice showed no decrease in lever pressing in the RI context but decreased responding in the RR context relative to the V day (one-sample *t*-test against 0.5: RI context *t*s’ < 0.83, *p*s’ > 0.4; RR context *t*s’ > 2.84, *p*s’ < 0.05). Once again GEE mice showed decreased lever pressing on DV compared to V days in both RI (*t*s’ = 5.97, *p*s’ < 0.01) and RR (*t*s’  = 6.98, *p*s’ < 0.01) contexts (Fig. 3d and Supplementary Fig. 2d).

Intriguingly, GEE altered lever press-associated activity of DLS MSNs during instrumental training and outcome devaluation testing. We first examined DLS MSN activity in the absence of lever-pressing, and found that GEE induced an increase in baseline firing rates of putative DLS MSNs (unpaired *t*-test: *t*_518_ = 2.18, *p* = 0.03) (Fig. 3c, e, f) (Supplementary Fig. 3c, d; “Methods”). We found evidence of lever-press related activity, with neurons increasing or decreasing their average firing rate during a ±2 s epoch around each lever press (Fig. 3c, e), in similar proportions between CE and GEE mice (Supplementary Fig. 3e). Early in training the proportion of putative DLS MSNs per mouse showing modulated firing rate around lever presses under both schedules was similar for putative DLS MSNs in GEE and CE mice (“Methods”) (*χ*^2^ = 0.36, *p* = 0.55) (Fig. 3g). This proportion was reduced in GEE mice late in training (*χ*^2^ = 5.60, *p* = 0.018) and during outcome devaluation (*χ*^2^ = 12.33, *p* = 0.0004) (Fig. 3g). These findings suggest that GEE results in increased basal activity of DLS MSNs but decreased engagement late in action learning and performance.

We next measured the magnitude of lever-press related firing rate modulation (both increases and decreases) of action-related DLS putative MSNs (Methods) and found that GEE altered rate modulation during lever-pressing in the RI context (ANOVA (Group × Day) interaction: F2, 188 = 4.93, *p* < 0.01) (Fig. 3h). In GEE mice, there was a net positive rate modulation during lever-pressing across training (one sample *t*-test against 0: *t*s’ > 2.94, *p*s’ < 0.01) that only emerged late in training in CE mice (Day 6: *t*_39_ = 1.90, *p* = 0.06). Furthermore, GEE resulted in significantly greater lever-press related modulation of DLS putative MSN activity at each point examined during RI schedule training (Day 1 unpaired *t*-test: *t*_69_ = 2.15, *p* < 0.05) (Day 6 unpaired *t*-test: *t*_58_ = 2.31, *p* = 0.06). Moreover, the strong decrease in lever pressing on the DV day in GEE mice observed in the RI context (Fig. 3d) was accompanied by a striking net negative modulation (Fig. 3h) (one sample t-test against 0: t_18_ = 2.40, *p* < 0.05) such that there was more net negative modulation of lever-press related DLS putative MSNs than observed in CE mice (*p* < 0.05). In contrast, there were no significant effects of GEE on the activity of these same DLS putative MSNs when the same mouse was tested in the RR context (Fig. 3i), arguing against sampling discrepancies as an explanation for changes in RI. GEE did not alter firing rate, recruitment, or modulation of lever-press related activity in DMS MSNs (Supplementary Fig. 3f–k).

### GEE results in greater excitability of DLS MSNs

We next determined if the GEE-induced dysfunctional cognitive phenotypes were accompanied by changes in intrinsic excitability and synaptic efficacy in DLS MSNs. We identified three potential mechanisms that could contribute to the GEE-induced behavioral phenotypes.

**Fig. 4:**
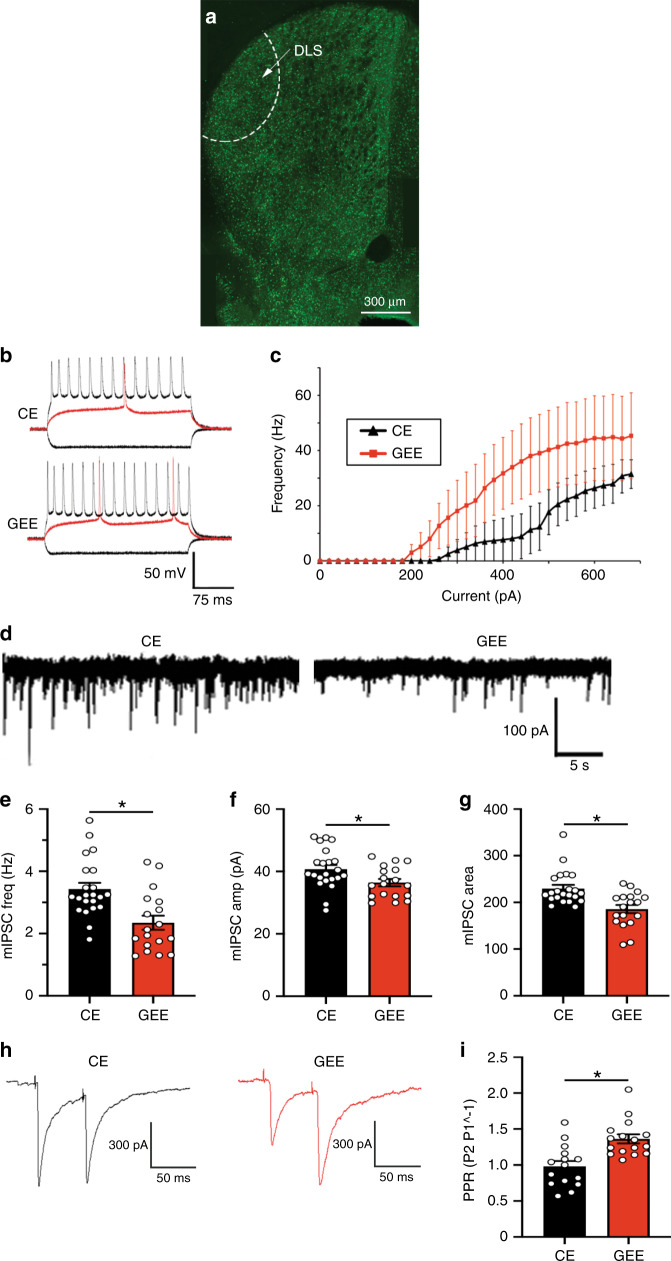
GEE increases MSN excitability and decreases GABAergic transmission onto DLS MSNs. **a** GAD65-GFP slice with the DLS demarcated. **b** Response of MSNs to hyperpolarizing (bottom black), just suprathreshold (middle red), and strongly depolarizing (top black) currents from CE (top), injected current = −400, +300, and +600 pA, respectively, and GEE (bottom) current = −400, +220 and +600 pA, respectively. **c** Current-AP frequency relationship shows an increase in the max frequency of AP in GEE compared to CE. **d** Representative mIPSCs recorded from CE and GEE MSNs. Graphs showing that mIPSC **e** frequency, **f** amplitude, and **g** area are decreased in GEE compared to CE. **h** Representative paired-pulse traces from CEE and GEE DLS-MSNs. **i** Paired-pulse ratio of DLS-MSNs recorded from CE and GEE mice with interstimulus interval of 50 ms. Error bars equal ± SEM, *t-test = *p* < 0.05.

First, using whole-cell recordings from DLS MSNs in adult brain slices (Fig. 4a), we observed a lower threshold for action potential firing and higher maximum firing frequency in response to intracellular current injection, indicative of greater excitability in GEE DLS MSNs compared to the CE group (repeated measures ANOVA (Group × Current step) interaction: F34, 306 = 1.80, *p* = 0.01; main effect (current step): F34, 306 = 15.71, *p* < 0.0001; no effect (Group): *p* = 0.16) (Fig. 4b, c). The input resistance, capacitance, resting membrane potential, and current-voltage relationship of DLS MSNs did not differ between groups (Supplementary Fig. 4). This GEE-induced increased intrinsic excitability could contribute to the net increase in DLS MSN firing observed with in vivo recordings.

### Altered GABAergic transmission onto DLS MSNs following GEE

Next, we examined GABAergic and glutamatergic miniature inhibitory and excitatory post-synaptic currents (mIPSCs and mEPSCs), respectively, in whole-cell recordings from adult GEE and CE DLS MSNs (Fig. 4a). The mIPSC frequency was decreased in GEE DLS MSNs compared to those from CE (unpaired *t*-test, *t*_38_ = 3.63 *p* = 0.0008) (Fig. 4d, e). The amplitude (unpaired t-test, *t*_38_ = 2.34; *p* = 0.02) and area (unpaired *t*-test, t_38_ = 3.78, *p* = 0.0005) of mIPSCs were also decreased in GEE mice (Fig. 4f, g). Increased paired-pulse ratio of electrical stimulation-evoked IPSCs in GEE DLS MSNs suggests decreased presynaptic release probability (unpaired *t*-test, *t*_29_ = 3.93; *p* = 0.0005) (Fig. 4h, i).

Interestingly, no differences were observed in glutamatergic mEPSC frequency or amplitude in DLS MSNs between groups (Supplementary Fig. 5), suggesting responses to incoming cortical or thalamic input are intact. Furthermore, no group differences were observed in GABAergic mIPSC frequency, amplitude and area in DMS MSNs (Supplementary Fig. 6), suggesting DS circuits supporting goal-directed strategies are left intact. Thus, GEE decreases both pre- and postsynaptic aspects of GABAergic synaptic transmission specifically onto DLS MSNs that support habitual decision-making.

### GEE decreases GABA release from PVs onto DLS MSNs

**Fig. 5:**
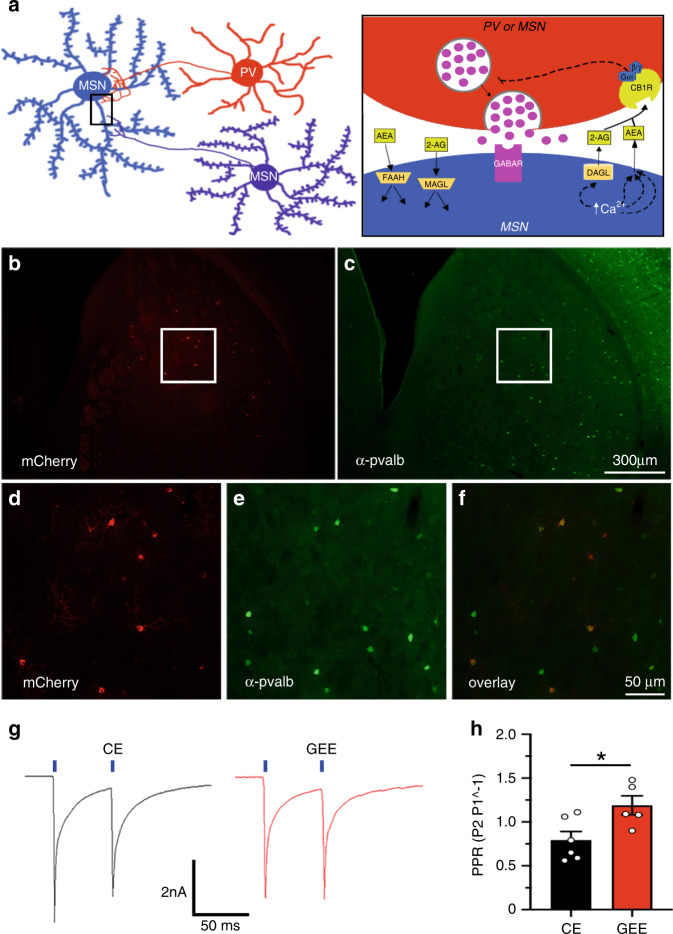
GEE decreases the efficacy of the parvalbumin-expressing interneuron to MSN synapse in the DLS. **a** Schematic diagram of the synaptic connectivity of PVs and MSNs (Left), and endocannabinoid siganling at the PV-MSN GABAergic synapse. **b**, **c** Example of a section obtained from a parvalbumin (*Pvalb*)-*cre* transgenic mouse injected into the dorsal striatum with a Cre-sensitive viral vector (*AAV2-DIO-ChR2-mCherry*) following immunohistochemistry using an antibody raised against against pvalb and visualization of mCherry. **d**–**f** Increased magnification of boxed regions in **b** and **c** shows that a majority of neurons that express mCherry also express pvalb. **g** Responses of MSNs recorded within DLS slices obtained from CE and GEE mice to two 5-ms pulses of 488 nm wavelength with an interstimulus interval of 50 ms. **h** Paired-pulse ratio of DLS-MSNs recorded from CE and GEE mice. Error bars equal ± SEM, *t-test = *p* < 0.05.

DLS MSNs receive GABAergic inputs from many sources (Fig. 5a)^36–38^. To explore GEE effects on specific DLS GABAergic inputs, we examined specific GABAergic synapses arising from one prominent source, the parvalbumin-expressing fast-spiking interneurons (PV). PVs form numerous feed-forward synapses onto the somata and proximal dendrites of MSNs^37^. Furthermore, PVs strongly contribute to mIPSCs in MSNs due to strong coupling ratios, synaptic transmission success rates, and prevalence of release sites compared to other GABAergic inputs^37^.

Therefore, we tested the hypothesis that GEE decreases the efficacy of PV-MSN synapses. We selectively expressed channelrhodopsin 2 (ChR2) using a Cre-sensitive viral vector (*AAV2-DIO-ChR2-mCherry*) in DS PVs of parvalbumin (*Pvalb*)-*cre* transgenic mice^39^ (Fig. 5b, c). We observed a significant increase in paired-pulse ratios of PV-driven oIPSCs recorded from GEE DLS-MSNs compared to CE (unpaired *t*-test, *t*_9_ = 2.66; *p* = 0.023) (Fig. 5d, e). This GEE-impairment at the PV-MSN synapse may contribute to the GEE decreased GABAergic transmission.

### DLS PV activation rescues GEE-induced lever-pressing rate

**Fig. 6:**
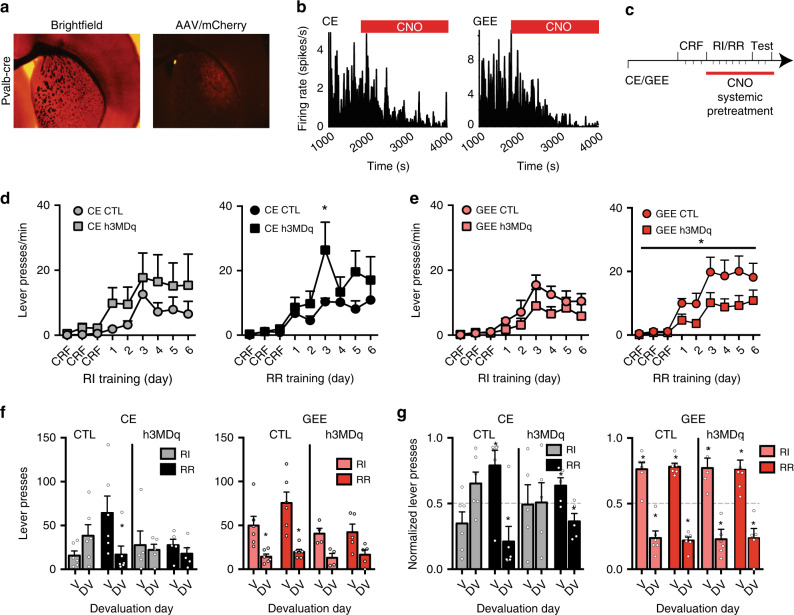
Chemogenetic activation of parvalbumin-expressing DLS interneurons rescues GEE-induced increases in lever-pressing rate. **a** Brightfield and fluorescent image of AAV/mCherry expression in the DLS of Pvalb-Cre mice. **b** Firing rate of DLS putative MSNs is decreased after i.p. injection of CNO in both CE (left panel) and GEE (right panel) mice. **c** Schematic diagram showing systemic pretreatment with CNO throughout RI/RR training and subsequent outcome devaluation testing. **d** Rate of lever-pressing during training under RI (gray) and RR (black) schedules in CE mice without (circle) and with (square) CNO activation of h3MD_q_ receptors. **e** Rate of lever-pressing during training in RI (pink) and RR (red) schedules in GEE mice without (circle) and with (square) CNO activation of h3MD_q_ receptors. Lever presses **f** and normalized lever-pressing **g** in RI and RR trained contexts during outcome revaluation testing in Valued (V) and Devalued (DV) states in CE and GEE mice with and without CNO activation of h3MD_q_ receptors. Error bars equal ± SEM, *p < 0.05.

We hypothesized that increasing PV activity may rescue the GEE-induced behavioral phenotypes. We thus took a chemogenetic approach with the designer receptor exclusively activated by the designer drug (DREADD), clozapine *N*-oxide (CNO)^40,41^. A cre-dependent viral vector expressing the G_q_-coupled hM3D_q_ DREADD or control vector virus was bilaterally injected into the DLS of CE and GEE *Pvalb*-*cre* mice (Fig. 6a; Supplementary Fig. 7a). Systemic CNO administration in awake-behaving mice decreased putative MSN firing, presumably through hM3D_q_-activation in PVs of CE and GEE mice (Fig. 6b; Supplementary Fig. 7b, c).

CE and GEE mice were given CNO daily prior to training and devaluation testing (Fig. 6c). Consistent with our previous observations, in the absence of h3MD_q_, GEE mice had a higher response rate under the RR schedule than CE mice (Group x Day interaction: F8, 80 = 1.91, *p* < 0.05), but not RI schedule (*p* > 0.5) (Fig. 6d, e; Supplementary Fig 7d, e). CNO selectively reduced response rates in the RR context of h3MD_q_–expressing PVs in GEE mice (Repeated measures ANOVA (Group x Day) interaction: F8, 80 = 1.94, *p* < 0.05) (Fig. 6e). In contrast, CNO increased response rates in the RR schedule for h3MD_q_-expressing CE mice (Repeated-measures ANOVA (Group x Day) interaction: F8, 72 = 3.10, *p* < 0.01) (Fig. 6d). CE and GEE mice expressing and not expressing h3MD_q_ showed similar response rates in the RI context, rewards and head entries (Supplementary Fig. 7f–i). Furthermore, CNO activation of h3MDq–expressing PVs had no effect on basal locomotor activity in CE or GEE mice (Supplementary Fig. 7k).

However, chemogenetic activation of DLS PVs did not restore use of habitual action strategies. During outcome devaluation testing, all GEE mice, both control vector- and hM3D_q_-expressing, decreased responding on the DV test day in both training contexts (main effect of Time: F1, 19 = 40.17, *p* < 0.0001; no interaction with or main effect of Group) (Bonferroni corrected *p*s’ < 0.05; Fig. 6f). In CE hM3D_q_-expressing and control vector mice, there was also a main effect of time (F1, 18 = 4.33, *p* = 0.05) (no interaction or main effect of group), but an unsupported follow-up analysis revealed that only CE mice injected with a control vector showed a reduction in responding in the DV test (Bonferroni corrected *p* < 0.05; Fig. 6f).

When we examined the normalized distribution of lever-presses between V and DV for each mouse (to account for differences in lever press rates), all CNO-treated GEE mice differently distributed their lever-presses between V and DV in both training contexts, independent of h3MD_q_-expressing PVs (one-sample t-test against 0.5: *t*s’ > 3.75, *p*s’ < 0.05) (Fig. 6g). Further, CNO-treated control vector- and h3MD_q_-expressing CE mice only differentially distributed their lever-pressing in the RR context (one-sample t-test against 0.5; *t*s’ > 2.5, *p*s’ < 0.06), but not RI context (*p*s’ > 0.1) (Fig. 6g). Thus, the GEE-induced decrease in DLS PV-MSN transmission contributes to the increased response rate phenotype, but not impaired decision-making.

### GEE-induced EC tone alters GABA transmission onto DLS MSNs

Additionally, it is possible that the decrease in mIPSC frequency in GEE DLS MSNs could result from altered neuromodulation. The neuromodulatory ECs decrease GABAergic synaptic transmission in DLS^42,43^, and are implicated in habit learning^44^. Cannabinoid receptor type 1 (CB1R) is highly expressed and functional in the developing DS, as early as E12.5 in rodents^45^. Alterations in either EC levels or CB1Rs may alter DLS GABAergic microcircuitry used during decision-making.

**Fig. 7:**
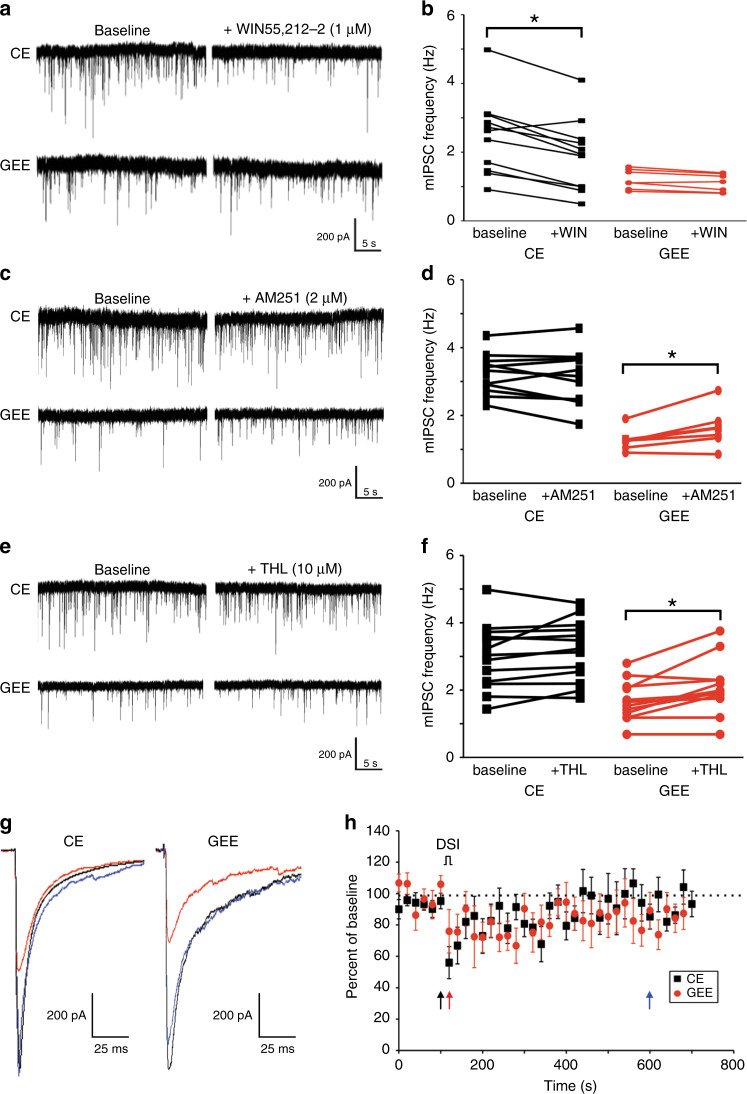
GEE alters tonic endocannabinoid modulation of GABAergic transmission that can be partially rescued by decreasing the endocannabinoid tone. **a** Representative traces of MSNs recorded from CE and GEE before (baseline) and during exposure to WIN55-212,2, a CB1R agonist. **b** Graph showing WIN55-212,2 effects on frequency of mIPSCs in CE (black) and GEE (red) mouse MSNs. Note the loss of agonist effect in GEE mouse neurons. **c** Representative traces of MSNs recorded from CE and GEE before (baseline) and during exposure to AM251, a CB1R antagonist. **d** AM251 increases the frequency of mIPSCs in GEE but not CE. **e** Representative traces of MSNs recorded from CE and GEE before (baseline) and during exposure to tetrahydrolipstatin (THL), a DAGL inhibitor. **f** Graph showing THL effects on mIPSCs frequency in CE and GEE mice, with increases only observed in GEE neurons. **g** Representative traces of MSNs recorded from CE and GEE before (black trace), after a 4-s membrane depolarization (DSI) (red trace) and ~8 min after DSI (blue tracE). **h** Graph showing DSI effects on mIPSC frequency in CE and GEE mice. The black, red and blue arrows denote the time at which the corresponding colored traces in g were taken. Error bars equal ± SEM, * = Repeated measures ANOVA Bonferroni corrected *p* < 0.05.

Thus, we tested the hypothesis that altered EC actions contribute to GEE effects on DLS GABAergic transmission. Acute application of the CB1R agonist WIN55,212 decreased mIPSC frequency in CE DLS MSNs, but not in GEE DLS MSNs (Repeated measures ANOVA (Group × Drug) interaction: F1, 16 = 11.05, *p* = 0.004; main effect Drug: F1, 16 = 23.73, *p* = 0.0002; main effect Group: F1, 16 = 6.13, *p* = 0.03) (Bonferroni corrected: CE mice *p* < 0.05; GEE mice *p* > 0.05) (Fig. 7a, b). Radioligand binding performed on the DLS of GEE and CE mice indicated no difference in agonist Bmax or Kd between the two groups, indicating that receptor levels were not broadly altered by GEE (Supplementary Fig. 8).

Excessive tonic CB1R signaling could depress mIPSC frequency, such that subsequent agonist application would have no further action. Bath application of the CB1R antagonist/inverse agonist AM251, increased mIPSC frequency in GEE DLS MSNs but not in CE, suggesting GEE CB1Rs are tonically active (Repeated measures ANOVA (Group × Drug) interaction: F1, 17 = 10.87, *p* = 0.004; no drug effect: *p* = 0.09; main effect Group: F1, 17 = 37.47, *p* < 0.001) (Bonferroni corrected CE mice: *p* < 0.05; GEE mice: *p* > 0.05) (Fig. 7c, d) and that GEE DLS MSNs can still be modulated by EC. Application of tetrahydrolipstatin (THL), an inhibitor of diacylglycerol lipase (biosynthetic enzyme for 2-arachidonoylglycerol (2-AG))^46,47^, increased mIPSC frequency in GEE, but not CE DLS MSNs(Repeated measures ANOVA (Group × Drug) no interaction: *p* = 0.13, main effect Drug: F1, 23 = 13.65, *p* = 0.001; main effect Group: F1, 23 = 13.91, *p* = 0.001) (Fig. 7e, f), suggesting that increased 2-AG contributes to the EC tone. However, this increased 2-AG level does not underlie the GEE-induced increase in MSN excitability, since THL did not alter excitability in CE or GEE DLS MSNs (Supplementary Fig. 9). There is currently no way to pharmacologically block anandamide (AEA) synthesis, as several pathways contribute to its production. Thus, we cannot rule out the possibility that increased AEA may also contribute to the GEE-induced increase in EC tone.

Depolarization-induced suppression of inhibition (DSI) of electrically-evoked IPSCs was similar in magnitude and time course between groups, suggesting that the observed increase in tonic EC does not affect phasic EC production, release or the ability of phasically released ECs to alter GABAergic transmission (two-way ANOVA, main effect time: F45, 835 = 2.12, *p* < 0.0001; no effect Treatment: *p* = 0.13; no effect interaction: *p* = 0.99) (Fig. 7g, h). This suggests that the pathway for CB1R modulation of GEE DLS MSNs is still intact, but tonic EC produces a constant, low-level suppression of GABAergic transmission.

**Fig. 8:**
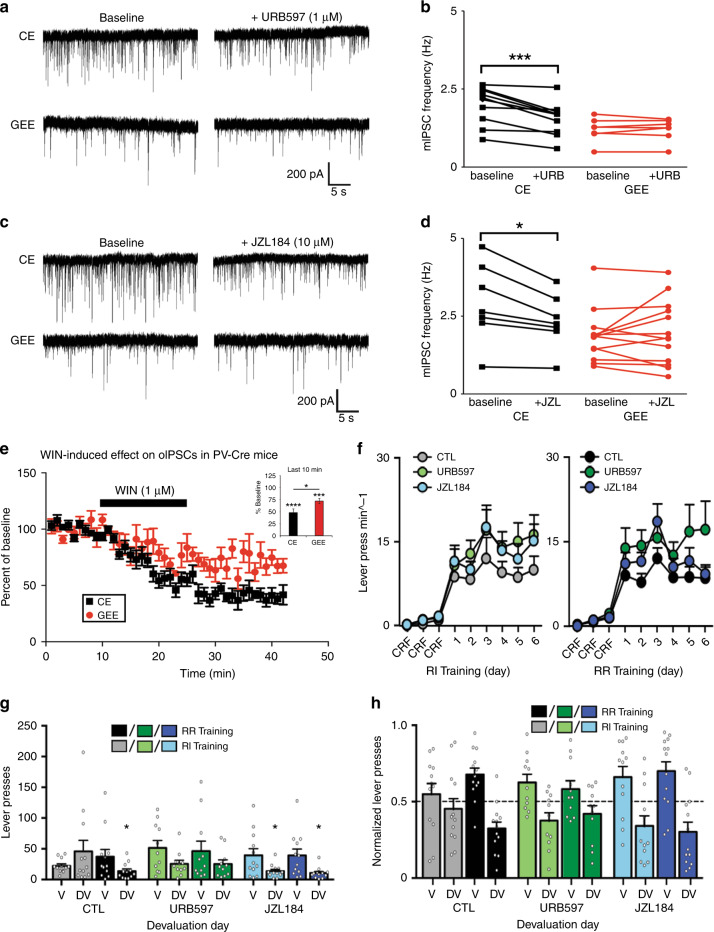
Pharmacological manipulation of endocannabinoid levels can mimic GEE effects. **a** Representative traces of MSNs recorded from CE and GEE before (baseline) and during exposure to URB597, a FAAH inhibitor. **b** Graph showing effects of URB597 on mIPSC frequency in MSNs from CE (black) and GEE (red) mice. Note that URB597 decreases frequency in CE but not in GEE MSNs. **c** Representative traces of MSNs recorded from CE and GEE before (baseline) and during exposure to JZL184, a MAGL inhibitor. **d** Exposure to JZL184 also decreases the frequency of mIPSCs recorded in CE MSNs but has no effect in GEE MSNs. **e** Effect of bath application of WIN on oIPSCs in PV-MSN synapse in CE and GEE DLS MSNs. Baseline was determined as the average oIPSC amplitude measured in the 10-minute window prior to WIN administration. Inset: average percent baseline measurements of oIPSCs over the last ten minutes of recording. **f** Response rate for pretreated mice under RI (left panel) and RR (right panel) schedules. **g** Lever-presses during drug-free outcome devaluation testing in valued (V) and devalued (DV) states. **h** Normalized lever-pressing during drug-free outcome devaluation testing in valued (V) and devalued (DV) states. Error bars equal ± SEM, *p < 0.05.

We therefore hypothesized that increasing EC tone would mimic GEE-induced inhibition of GABAergic transmission. EC signaling is tightly regulated by enzymatic hydrolysis, with fatty acid amide hydrolase (FAAH) and monoacylglycerol lipase (MAGL) catalyzing the degradation of AEA and 2-AG, respectively^48,49^. Bath application of either URB597 or JZL184 (Fig. 8a–d), inhibitors of FAAH or MAGL respectively, decreased mIPSC frequency in CE but not GEE DLS MSNs (URB597 Repeated measures ANOVA (Group × Drug) interaction: F1, 15 = 14.10, *p* = 0.002; main effect Drug: F1, 15 = 13.70, *p* = 0.002; main effect Group: F1, 15 = 5.68, *p* = 0.03; Bonferroni corrected: CE mice *p* < 0.05, GEE mice *p* > 0.05) (Fig. 8a, b) (JZL184 Repeated measures ANOVA (Group × Drug) interaction: F1, 18 = 7.74, *p* = 0.012; no effect Group: *p* = 0.08; no effect Drug: *p* = 0.12; Bonferroni corrected: CE mice *p* < 0.05; GEE mice *p* > 0.05) (Fig. 8c, d). Increasing ECs in CE mice partially mimics GEE effects on GABAergic synapses suggesting that EC tone in GEE mice interferes with tonic CB1R activation that could occur under constant release of low levels of ECs.

We then tested the hypothesis that the GEE-induced decrease in the efficacy of the PV-MSN synapse involves altered EC. To this end, we once again selectively expressed ChR2 into DS PVs using the *Pvalb*-*cre* transgenic mice and recorded oIPSCs from DLS MSNs in control and GEE mice before, during, and after bath application of the CB1R agonist, WIN 55,212-2 (1 μM). WIN induced a long-lasting reduction of oIPSC amplitude in DLS MSNs in both CE (48.5 ± 7.3% of baseline; *t*_12_ = 6.99, *p* < 0.0001) and GEE mice (72.2 ± 5.3%; *t*_10_ = 5.30, *p* = 0.0003) (Fig. 8e), but greater depression was observed in CE MSNs (t_11_ = 2.54, *p* = 0.03). These data indicate that EC signaling is dysregulated at PV-MSN synapses, likely contributing to the alteration in mEPSC frequency.

### Increasing EC tone mimics GEE-induced cognitive deficits

If increased EC tone suppresses GABAergic transmission similar to GEE, then it might also mimic the dysfunction in habit learning. Adult naïve mice were given systemic injections of URB597 (FAAH inhibitor), JZL184 (MAGL inhibitor), or vehicle^48^ 2 h prior to RI and RR schedule training for 6 days (Supplementary Fig. 10a). Increasing EC tone did not significantly alter acquisition or rate of lever-pressing in either context, although it appeared slightly increased (repeated measures ANOVA (Treatment x Training day) interaction: *F*s < 1.65, *p*s > 0.05). Mice similarly increased response rate and lever-pressing across RI and RR training (main effect of Training day: *F*s > 37.85, *p*s < 0.01) (Fig. 8f; Supplementary Fig. 10b), and there were no differences in rewards earned or head entries (Supplementary Fig. 10c, d).

Following outcome devaluation, vehicle-treated mice reduced lever-pressing in the RR but not RI context (ANOVA (Schedule × Revaluation state) interaction: F1, 23 = 3.38, *p* < 0.05) (Bonferroni corrected RR context *p* < 0.05) (Fig. 8g). Vehicle-treated mice also differently distributed their lever-pressing in the RR, not RI context (one-sample t-test against 0.5: RR t_11_ = 3.94, *p*s’ < 0.01; RI *p* > 0.05) (Fig. 8h) (Supplementary Fig. 10e). However, following outcome devaluation URB597- or JZL184-treated mice reduced lever pressing in both RI and RR contexts (URB597 Repeated measures ANOVA (Schedule × Revaluation state): no interaction; main effect of Revaluation state: F1, 20 = 6.97, *p* < 0.05) (JZL184 Repeated measures ANOVA: no interaction; main effect of Revaluation state: F1, 23 = 14.03, *p* < 0.01; Bonferroni corrected RI and RR *p*s’ < 0.05) (Fig. 8g), and differently distributed their lever-pressing in both RI and RR training contexts ((one-sample t-tests against 0.5) URB597: RI *t*_10_s’ = 2.30, *p*s’ < 0.05; RR *t*_10_s’ = 1.95, *p*s’ = 0.07; JZL184: RI *t*_11_s’ = 2.28, *p*s’ < 0.05; RR *t*_11_s’ = 3.04, *p*s’ < 0.05) (Fig. 8h) (Supplementary Fig. 10e). Head entries were only reduced in vehicle-injected mice in DV (Supplementary Fig. 10f). Consumption of either pellets or sucrose directly prior to revaluation testing was similar between groups (Supplementary Fig. 10g). Thus, increasing EC tone is sufficient to reproduce GEE impaired habits, implicating EC dysregulation as a mechanism for GEE–induced disruption to decision-making strategies.

## Discussion

Efficient action performance is advantageous for daily living, as the constant use of goal-directed processes can involve unnecessary expenditure of cognitive resources. The observed GEE-induced behavioral disruptions resemble human FASD perturbations in cognition and action performance, including processing speed^1,4^. It is noteworthy that GEE mice were deficient in behavioral automatization, relying more on functionally demanding cognitive processes. Although deficits in these operationally defined action strategies have not yet been evaluated in human FASD, our findings suggest their potential as biobehavioral markers^50^.

Extensive evidence supports the idea that varying degrees of behavioral and physical anomalies associated with FAE are dependent on the dose, pattern and timing of the alcohol insult relative to the development of a given brain region^51,52^. Our noninvasive GEE vapor model produced near-intoxicating chronic EtOH exposure levels throughout a period equivalent to three trimesters of human brain development^26,27^ (Fig. 1). Exposure to the vapor apparatus itself did not underlie the observed effects (no differences between CE and naïve mice, Supplementary Fig. 11). Indeed, we did not observe gross physical deformities typical of FAS or mice after higher dose exposure^1^. Previous studies showed that FASD results in reduced putamen (DLS) volume^5^ that correlated with lower IQ and severity of symptoms^53^. Thus, impairment in the putamen/DLS appears to play an important part in FASD. Although we did not observe DMS dysfunction, human studies have also found reduced caudate (DMS) volumes^5,53^, that may also contribute to disrupted cognitive function in FASD.

The GEE-induced physiological disruptions suggest that DLS MSNs would display enhanced responsivity to cortical input. It is worth noting that higher prenatal EtOH doses were  reported to alter corticostriatal transmission, indicating that additional mechanisms can contribute to changes in MSN function^54^. While others have explored interactions between ECs and alcohol^55^, less is known about their interaction during development^56,57^. The selectivity of GEE-induced EC tone for GABAergic versus glutamatergic synapses likely reflects the higher EC sensitivity of striatal GABAergic synapses^58^. The GEE-induced increase in EC tone contributed to decreased GABA release but not to postsynaptic GABAergic changes as pharmacological manipulations in EC altered mIPSC frequency and not other mIPSC characteristics (Supplementary Table 1). Both ECs and GABA play important roles throughout brain development^59–62^. In addition to the pharmacological effects of EtOH itself, the stress of drug exposure and withdrawal might also contribute to these changes, as stress has profound effects on the brain EC system^63^. Technical limitations prevented our ability to alter EC tone at selected synapses (i.e. PV-MSN) in vivo to determine whether EC dysregulation at the PV-MSN synapse contributes to these phenotypes. Further, systemic disruption of EC degradation at other brain regions may contribute to the GEE-altered cognitive function. Additionally, we did not determine if GEE produced differential effects on direct (D1-MSNs) versus indirect (D2-MSNs) pathway neurons in the DLS. It has been demonstrated that D1-MSNs receive stronger glutamatergic inputs than D2-MSNs in the in vivo DLS^64^. Therefore, the GEE-decrease in DLS GABAergic transmission may affect dMSNs more than iMSNs, and this imbalance might underlie the observed increase in lever pressing or strong devaluation. The recent findings that GEE caused a long-term increase in glutamatergic transmission onto DMS D1-MSNs^65^ and increased activity of DLS neurons^66^ indicates another mechanism that may contribute to the observed behavioral phenotypes.

In summary, we identify multiple mechanisms through which GEE disrupts cognitive function via DS inhibitory microcircuits, a brain region implicated in human FASD that mirror cognitive dysfunction observed in human FASD, and may prove useful in developing behavioral biomarkers for FASD. These results provide a greater understanding of the molecules, cells and circuits involved in FASD behavioral disruptions and open the door for novel potential therapeutic strategies for FASD treatment.

## Methods

### Ethanol administration

All experiments were approved and conducted in accordance with the guidelines from the National Institute on Alcohol Abuse and Alcoholism (NIAAA) Animal Care and Use Committee, and approved by this committee. Mice were housed on a 12 h light/dark cycle (0630-1830 light) with mouse chow and water ad libitum. Transgenic mice (gGAD65_3e/gfp3.3)#15) expressing enhanced green fluorescent protein (eGFP) under the GAD65 promoter on a C57Bl/6J background, generated as described previously^67^, were obtained from The Jackson Laboratory (Bar Harbor, ME), and bred in-house (referred to as GAD65-GFP). Additionally, transgenic parvalbumin (*Pvalb*)-*cre* mice^39^ were obtained and bred in-house.

Cohorts of mice were exposed to the vapor procedure. Female C57Bl/6J mice obtained from The Jackson Laboratory were mated with GAD65-GFP or *Pvalb-cre* males. Additionally, *Pvalb-cre* females were mated to *Pvalb-cre* males. Upon appearance of a vaginal plug, pregnant dams were single or pair housed and placed into either an ethanol vapor or control (air) vapor group. Dams were placed in their respective chamber and exposed to ethanol vapor or air for 16 h/day, 4 days/week from E0.5-P10 (Fig. 1a), similar to the chronic intermittent exposure protocol previously described^68^. At the beginning of each session, dams and food were weighed and then transferred to a new polycarbonate cage that contained only mouse bedding. The lid of the cage was removed to allow for easy access to the air or ethanol-vapor. At the end of the session, body weight and food consumed were measured. Dams were returned to their home cage.

The rate at which ethanol was volatilized by a high capacity pressure pump (Cole-Parmer, Vernon Hill, IL) determined the concentration of ethanol that was delivered to the chamber. Air and ethanol-vapor were delivered to their respective chambers at a rate of 10 liters per minute (LPM). During pregnancy (E0.5-~E21.5), the concentration of ethanol vapor at the beginning of the session averaged 200 mg/dl and averaged 150 mg/dl at the end of the session. Mothers and litters (P0-P10) were exposed to 100 mg/dl of ethanol at the beginning of the session and averaged 60 mg/dl at the end of the session. This elicited no detectable BEC in the mothers. However, since the activity of the enzyme, alcohol dehydrogenase, does not reach adult levels until the third postnatal week in rodents^68–70^, the average BEC in these pups were statistically similar to what was detected in dams during pregnancy using this paradigm (Fig. 1b). This allowed for the exposure of ethanol to pups without maternal separation and the lack of a detectable BEC in mothers allowed them to properly care for their young as previously demonstrated, and as evidenced by proper nesting behavior and the presence of a milk spot in their neonates.

Blood was collected via tail nicks once a week in at least one CE and one GEE mouse per cohort, to measure BEC. BEC was measured using a GM7 analyzer (Analox Instruments, Lunenburg, MA). Briefly, ethanol was oxidized by the enzyme alcohol oxidase in the presence of molecular oxygen to acetylaldehyde and hydrogen peroxide. A Clark-type amperometric oxygen electrode monitored the rate of oxygen consumption, which is directly proportional to the concentration of ethanol. Litters were counted and weighed daily from P0–P10. BECs were measured in pups via trunk blood collected at the time of sacrifice. BEC was expressed in mg/dl.

For all other experiments, male and female offspring from the GAD65 breeding pairs, as well as male C57Bl/6J mice purchased from The Jackson Laboratory of at least 8 weeks old were used. Mice used for behavioral and brain slice electrophysiological experiments were housed in groups of 1–4; mice used in recording experiments were singly housed after surgical procedures. Mice were kept on a 12 h light/dark cycle, and all recording and behavioral experiments were performed during the light portion of the cycle.

### Instrumental food training

For the initial instrumental behavioral experiments, a total of *n* = 11 CE and *n* = 11 GEE mice from four exposure cohorts were used. For the in vivo recording instrumental experiment, *n* = 5 CE and *n* = 6 GEE mice from three vapor cohorts were used. For the hM3Dq activation in DLS Parv interneurons, *n* = 16 CE, and *n* = 16 GEE mice from two cohorts were used. For the comparison to naïve non-vapor exposed mice, a total of *n* = 4 naïve, *n* = 4 CE, and *n* = 8 GEE mice from two cohorts were used. All behavioral training and testing took place as previously described^14^. In brief, mice were placed in operant chambers in sound attenuating boxes (Med-Associates, St. Albans, VT) in which they pressed a single lever (left or right) for an outcome of either regular “chow” pellets (20 mg pellet per reinforcer, Bio-Serve formula F05684) or sucrose solution (20–30 μl of 20% solution per reinforcer). The other outcome was provided later in their home-cage and used as a control for general satiation in the outcome devaluation test. Before training commenced, mice were food restricted to 90% of their baseline weight at which they were maintained for the duration of experimental procedures.

As previously described for the within-subject design^14^, training was conducted as follows: each day each mouse was trained in two separate operant chambers distinguished by contextual cues [i.e. black and white vertical striped laminated paper on chamber walls (3.2 mm wide stripes) or clear plexi-glass chamber walls]. Upon completion of training in one context, mice were immediately trained in the remaining context. For each mouse, the order of schedule exposure, lever position and the outcome obtained upon lever pressing were kept constant across contexts. However, mice were counterbalanced for context, schedule order, lever position, and outcome earned. Each training session commenced with illumination of the house light and lever extension, and ended following schedule completion or after 60 min with the lever retracting and the house-light turning off.

On the first day, mice were trained to approach the food magazine (no lever present) in each context on a random time (RT) schedule, with a reinforcer delivered on average every 60 s for a total of 15 min. Next, mice were trained in each context on continuous reinforcement schedules (CRF), where every lever-press made was reinforced, with the possible number of earned reinforcers increasing across training days (CRF5, 15, 30). In the absence of any predictive cue signaling reward delivery, unimplanted mice acquired lever-press behavior with 3 days of CRF, while mice used in the recording experiment took on average 6 ± 1 days of CRF training (CRF5, 15, 30 × 4) to press the lever consistently, with no difference between CE and GEE groups in either implanted or unimplanted mice. After acquiring lever-press behavior, mice were trained on random interval (RI) and random ratio (RR) schedules of reinforcement with schedules differentiated by context, and the possibility of earning 15 reinforcers in each context or until 60 min had elapsed. Mice initially pressed under RI30 (on average one reinforcer following the first press after an average of 30 s) and RR10 (on average one reinforcer every 10 lever presses) schedules for two days, followed by four days of RI60 and RR20 training.

Outcome devaluation testing occurred across two consecutive days, with testing occurring in each context. In brief, on the valued day, mice had ad libitum access to the home-cage outcome for 1 h before serial brief non-reinforced test sessions in the previous RI and RR training contexts. On the devalued day, mice were given 1 h ad libitum access to the outcome previously earned by lever-press, and then underwent serial non-reinforced test sessions in each training context. Pre-feeding took place in a separate cage to which mice were previously habituated, and the amount consumed was recorded. Order of context exposure during testing was the same as training exposure, with order of devaluation day counterbalanced across mice. Tests in each context were either 10 min (recording mice) or 5 min in duration.

For mice trained only on the RI training schedule, training and devaluation testing proceeded exactly as for mice in the within-subject design (RI and RR schedule training), except that mice were only trained on the RI schedule in one context. Additionally, to equate for the total number of possible reinforcers earned, mice had the opportunity to earn 30 reinforcers or remain in the chamber until 60 min had elapsed during the RI training.

### Locomotor activity in a novel cage

Mice (*n* = 12 CE, 14 GEE) were placed in a novel polycarbonate cage similar to those used in the vapor chambers, for 20 min a day for three consecutive days. Horizontal activity was detected as infrared beam crosses (1 inch spacing, 10 beams per cage) within 10-s bins using Opto M3 activity monitors (Columbus Instruments, Columbus, OH). Once the trial was over, mice were immediately returned to their home cage. Data were expressed as average number of infrared beam breaks per minute ± SEM.

### Systemic administration of endocannabinoid degradation inhibitors during schedule acquisition

To increase endocannabinoid levels^49^ during RI and RR schedule training, vapor-naïve mice were given an i.p. injection 2 h prior to each training day (6 days) of the FAAH inhibitor URB597 (*n* = 11) (10 mg/kg) (10 ml/kg), the MAGL inhibitor JZL184 (*n* = 13) (16 mg/kg) (10 ml/kg), or vehicle (10 ml/kg) (*n* = 12). To control for pretreatment injection effects, saline pretreatment (10 ml/kg) injections were given on RT, CRF, and outcome devaluation days. Drugs were dissolved in DMSO and Cremophor, and brought to final concentrations with saline at a 1:1:18 ratio respectively.

### Expression of channelrhodopsin 2 into the DS of parvalbumin-expressing interneurons of CE and GEE mice

Parvalbumin (*Pvalb*)-*cre* transgenic mice on a C57Bl/6J background were mated. Upon appearance of a seminal plug, mice underwent either the CE or GEE paradigm. To express the light-activated cation channel, channelrhodopsin 2, specifically in parvalbumin-expressing interneurons, a cre-inducible AAV-***hSyn***-DIO-ChR2-mcherry (University of Pennsylvania Vector Core) was infused bilaterally into DS (B: 0.5 mm, ML: 2.20 mm, and V: −3.50 mm) of 8-week-old CE and GEE parvalbumin (*Pvalb*)-*cre* transgenic mice. The DS was stereotaxically targeted, with virus (200 nl) infused via manual compression of a Hamilton syringe at a rate of 20 nl/min. The syringe was left in place for an additional 7–10 min to allow for diffusion away from the injection site. At least 2 weeks following injection, ChR2-expressing mice (CE *n* = 8; GEE *n* = 4) (3 cohorts) were sacrificed for electrophysiological analysis. MSNs were targeted for electrophysiological recording. A 5-ms pulse of 488 nm wavelength LED (Thor Labs) was used to activate ChR2-positive parvalbumin-expressing interneurons.

### Chemogenetic activation of DLS parvalbumin interneurons during acquisition and devaluation testing

For chemogenetic activation of parvalbumin interneurons, a cre-inducible AAV-hSyn-DIO-hM3D_q_-mcherry (Gene Therapy Vector Core at the University of North Carolina) was infused bilaterally into DLS (B: 0.5 mm, ML: 2.30 mm, and V: −3.00 mm) of parvalbumin (*Pvalb*)-*cre* transgenic mice and their wild-type littermates. The DLS was stereotaxically targeted, with virus (300 nl) infused via manual compression of a Hamilton syringe at a rate of 20 nl/30 s. The syringe was left in place for an additional 7–10 min to allow for diffusion away from the injection site. Three weeks following injection, hM3D_q_ (CE *n* = 8; GEE *n* = 9) and control (CE *n* = 8; GEE *n* = 7) (2 cohorts) mice were trained using the within-subject design. During acquisition and outcome devaluation testing, mice were given a 1-h pretreatment with clozapine-n-oxide (CNO) (1 mg kg^−1^) (10 ml kg^−1^) before operant procedures. To confirm hM3D_q_ activity, we implanted an electrode array at the site of virus infusion in a subset of mice. Firing rate of putative MSNs (see below) was assessed for +1 h after CNO or saline injection relative to the preceding drug-free baseline-firing rate (Figure X; Supplementary Fig. X). The effect of hM3D_q_ activation on locomotor activity was assessed 2-weeks post completion of operant training and devaluation testing. Mice were given pretreatment with CNO (1 mg kg^−1^) (10 ml kg^−1^) 1 hour prior to assessment, and then placed in clear polycarbonate cages (10.25 × 6 inches), and horizontal activity was detected as infrared beam crosses (1-inch spacing, ten beams per cage) made on consecutive beams (ambulatory counts) using Opto M3 activity monitors (Columbus Instruments). Data were expressed as average number of infrared beam breaks 5 min bin ± SEM. Virus spread was assessed under a fluorescence microscope, and mice were excluded for extensive spread into surrounding cortices. Final *n*’s were the following; CE Ctl *n* = 6, CE hM3D_q_ *n* = 5, GEE Ctl *n* = 6, GEE hM3D_q_ *n* = 5.

In vivo extracellular recordings: as previously described, mice (*n* = 5 CE, *n* = 6 GEE) (3 cohorts) were implanted with multi-electrode arrays for in vivo recordings of neural activity during awake behavior^11,14^. Mice were implanted with one or two arrays, targeting the DLS and DMS in one or both hemispheres. Two rows of eight electrodes (platinum-coated tungsten, 50 μm, CD Neural), with electrodes spaced 200 μm apart and rows spaced 1.2 mm apart targeted the DLS and DMS. Arrays were centered 0.5 mm anterior and 1.75 mm lateral to the bregma, and then lowered 2.2–2.4 mm from the surface of the brain. Upon experiment completion, mice were perfused and brains fixed with 4% w/v paraformaldehyde, and array placement was verified using Nissl-stained brain slices (50 μm). All mice had electrode tracks within the DLS and DMS.

### Neuronal recordings during behavior

Mice were allowed at least 2 weeks of recovery before the start of behavioral and recording procedures. In brief, spike activity was recorded using the MAP system (Plexon Inc., TX) and initially sorted using an online-sorting algorithm. Mice were moved from one context to the other without disconnecting the headstage, and the same online sorting algorithm was used in both contexts on the same day. To synchronize the recordings with lever-press behavior, we used TTL pulses sent from a Med-Associates interface board to the MAP recording system through an A/D board (Texas Instruments Inc., TX) to behaviorally timestamp the neural activity (10 ms resolution of the behavior). Data were then resorted offline (Offline Sorter, Plexon, Inc.) to identify single unit neuronal activity based on waveform, amplitude, and interspike interval histograms (no spikes during a refractory period of 1.3 ms). Units with a half-width of <100 μs and baseline firing rate more than 10 hz, as well as units with a waveform trough half-width more than 250 μs were excluded; the remaining units were classified as putative MSNs^11^.

### Lever-press related neuronal activity during training and outcome devaluation

To examine lever-press-related neural activity in both RI and RR training and testing contexts, for each previously isolated recorded unit we constructed a peri-event histogram (PETH) around time-stamped lever-presses, where neural activity was averaged in 20-ms bins, shifted by 1 ms and averaged across trials to analyze amplitude and latency around the recorded behaviors. Using the distribution of the PETH from 5 to 2 s before the lever-press as baseline activity, we slid 1 ms steps across 20-ms bins from 2 s before to 2 s after lever-press events. We identified a lever-press-related neuron as a unit with a significant change in firing rate within this window in two ways. A lever-press related neuron was up-modulated if it had a significant increase in firing rate defined as at least 20 consecutive overlapping bins with a firing rate higher than a threshold of 99% above baseline activity. A lever-press related neuron was down-modulated if it had a significant decrease in firing rate defined as at least 20 consecutive bins with a firing rate lower than a threshold of 95% below baseline activity^11,14^. The onset of lever-press related activity was defined as the first of these 20 consecutive significant bins. Rate modulation was calculated for each unit as the mean frequency during the significant modulation bins/mean baseline frequency. Only neurons that modulated firing rate during lever-pressing in both RI and RR contexts were included in analyses^14^.

### Slice preparation and electrophysiology

Mice were anesthetized with isoflurane and decapitated. Brains were removed and placed in ice-cold cutting solution containing in mM: sucrose 194, NaCl 30, KCl 4.5, NaHCO_3_ 26, NaH_2_PO_4_ 1.2, glucose 10. Coronal brain slices containing the dorsal striatum, 250-μm thick were obtained using a vibrating blade microtome (Leica VT1200S) and recovered in aerated ACSF containing in mM: NaCl 124, KCl 4.5, MgCl_2_ 1, NaHCO_3_ 26, NaH_2_PO_4_ 1.2, D-glucose 10, CaCl_2_ 2 at 33 °C for 1 hour. Slices were then placed at room temperature until experimental use. Whole-cell patch clamp recordings were performed between 28 and 30 °C ± 1 °C (with control by an Automatic Temperature Controller, Warner Instruments, Hamden, CT). Neurons in slices were visualized with an upright microscope using a 40× (0.8 n.a.) water immersion objective. Real-time images were displayed on a video monitor, which aided navigation and placement of recording pipettes. Patch pipettes were pulled from borosilicate glass capillaries (1.5 mm outer diameter, 0.86 mm inner diameter; World Precision Instruments, Sarasota, FL) and filled with internal solution. Two internal solutions were used. The K-based internal contained in mM: K-gluconate 126, KCl 4, HEPES 10, Mg-ATP 4, Na-GTP 0.3, Phosphocreatine 10. The Cs-based internal contained in mM: CsCl 150, HEPES 10, MgCl_2_ 2, Na-GTP 0.3, Mg-ATP 3, BAPTA-4Cs 0.2. When filled with internal solution, the patch pipettes had resistances of 2–4 MΩ. Recordings were made using a Multiclamp 700 A amplifier (Molecular Devices, Foster City, CA). Membrane currents were filtered at 2 kHz, digitized using a Digidata 1322 A at 10 kHz, displayed and saved using Clampex v9.2, and analyzed with Clampfit v9.2 (Molecular Devices) or MiniAnalysis (Synaptosoft v6.0.7, Decatur, GA). Statistical analysis was performed using SigmaStat 3.0 (SPSS Inc., Chicago, IL) or GraphPad Prism 5 (GraphPad Software, Inc. LaJolla, CA). Data were reported as mean ± SEM. The following drugs were routinely used to isolate mIPSCs (*n* = 22 CE mice from 13 cohorts, *n* = 18 GEE mice from eight cohorts: APV (50μM, Tocris), NBQX (5 μM, Tocris), tetrodotoxin (TTX, 1 μM, Tocris). To isolate mEPSCs, picrotoxin (100 μM, Sigma) and tetrodotoxin (TTX, 1 μM, Tocris) were used (*n* = 5 CE mice from three cohorts, *n* = 5 GEE mice from three cohorts). The following drugs were acutely applied during whole-cell patch clamp electrophysiology experiments to examine different aspects of the endocannabinoid system: AM251 (2 μM, Tocris; *n* = 5 CE mice from 3 cohorts, *n* = 4 GEE mice from three cohorts), WIN55,212 (1 μM, Tocris; *n* = 5 CE mice from three cohorts, *n* = 5 GEE mice from three cohorts), THL (10 μM, Tocris; *n* = 5 CE mice from 3 cohorts, *n* = 4 GEE mice from three cohorts), JZL184 (10 μM, Tocris; *n* = 5 CE mice from three cohorts, *n* = 6 GEE mice from three cohorts), URB597 (1 μM, Tocris; *n* = 5 CE mice from three cohorts, *n* = 6 GEE mice from three cohorts).

### Statistical analyses

The α level was set at 0.05 for all analyses, unless otherwise indicated. Initial analyses showed normal distributions for all behavioral data. For all behavioral analyses, lever presses, lever press rate, rewards earned, and head entries, as well as drug-treatment were analyzed using repeated measures ANOVA, with post-hoc analyses performed using Bonferroni-corrected paired *t*-tests where appropriate. For outcome devaluation testing analyses, two-way ANOVA (Devaluation state × Schedule) within each exposure group (GEE or CE) and Treatment group (Ctl, URB597, or JZL184) (Ctl or hM3D_q_) were used to evaluate differences in lever-press and consumption behavior with post-hoc analyses performed using Bonferroni-corrected paired *t*-tests where appropriate. To investigate the within-subject distribution of lever-presses between Valued and Devalued states, we normalized lever-presses for Valued and Devalued states to total lever-pressing (Valued + Devalued) in each context. We then conducted one-sample *t*-tests for normalized data to examine whether each condition differed from chance (0.5); that is, what distribution of lever presses between Valued and Deavlued states for each schedule was observed in normalized data, with a value of 0.5 reflecting the same level of lever pressing between Valued and Devalued states. Additionally, we examined the magnitude of outcome devaluation by creating a devaluation index ((lever presses Valued state—lever presses Devalued state)/(lever presses Valued state + lever presses Devalued state)) for each mouse in the RR and RI contexts. We then conducted paired *t*-tests to examine differences in the magnitude of devaluation between RI and RR contexts.

For the analyses of in vivo physiological data, paired t-tests and 2-way ANOVAs (Bonferroni-corrected) were used to assess exposure-induced differences in firing rate and rate modulation. One-sample t-tests against zero were used to examine significant positive or negative rate modulation changes. Chi square tests were performed to evaluate proportional differences in lever-press related activity per mouse. Data analyses were performed using Neuroexplorer, Graphpad Prism, and Matlab (Mathworks).

### Reporting summary

Further information on research design is available in the Nature Research Reporting Summary linked to this article.

## Supplementary information

Supplementary Information

Reporting Summary

## Data Availability

The data that support the findings of this study are available from the corresponding author upon reasonable request. Source data underlying the figures are available as a Source Data file.

## Source data

Source Data
