# Supplementary material for: Gestational alcohol exposure disrupts cognitive function and striatal circuits in adult offspring
Source: Nat Commun. 2020 May 22;11:2555. doi: 10.1038/s41467-020-16385-4 (PMC7244532; doi:10.1038/s41467-020-16385-4)
Supplement: Supplementary file 1 — Supplementary Information [file 41467_2020_16385_MOESM1_ESM.pdf]

Supplementary Information

Gestational alcohol exposure disrupts cognitive function and striatal circuits

Cuzon Carlson et al.

SUPPLEMENTARY FIGURES

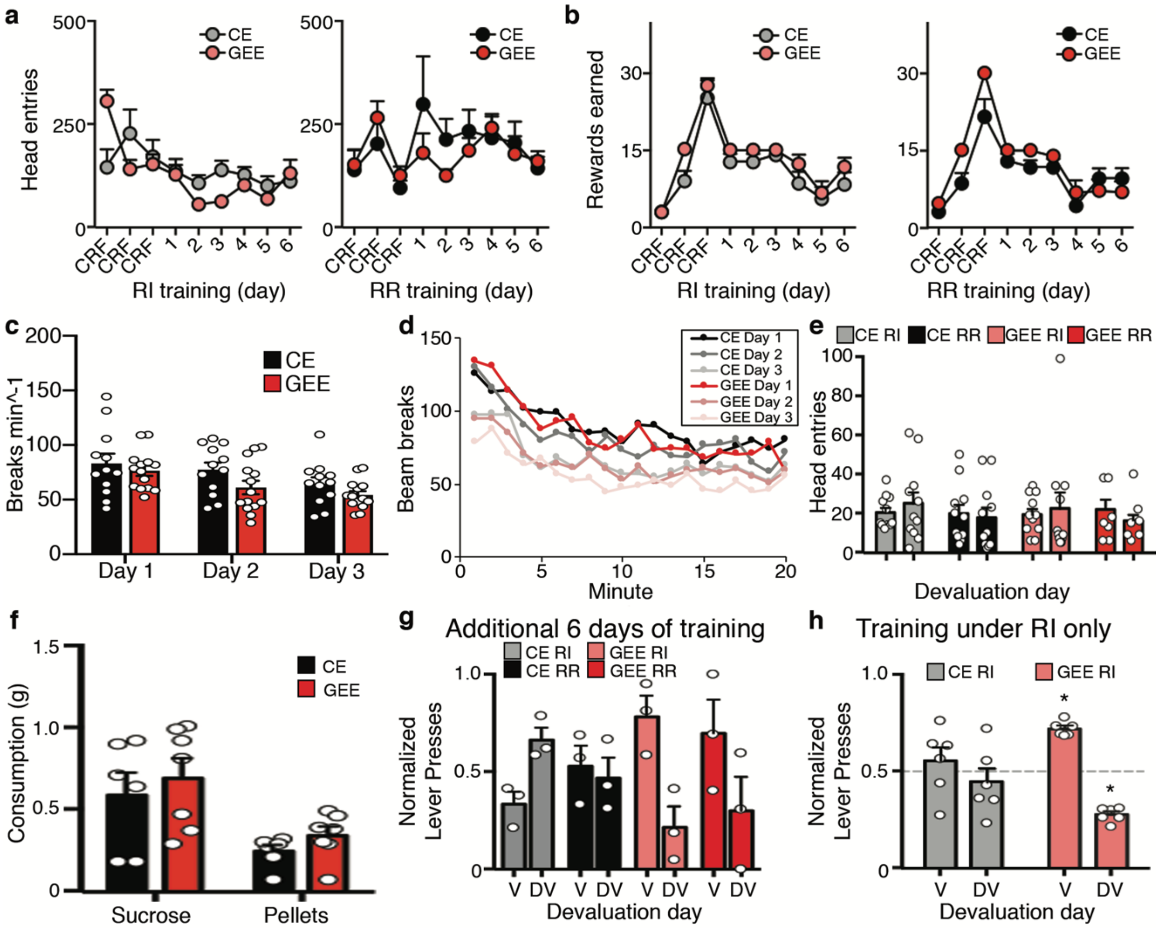

Supplementary Figure 1. Effects of GEE on acquisition and execution of goal-directed and habitual actions. Mice were exposed to air (CE; n = 11) or ethanol (GEE; n = 7) via vapor inhalation through gestation (E0.5 – P10). At 2-3 months of age, mice underwent instrumental lever-press training using a within-subject design. (**a**) A repeated-measures ANOVA (Exposure x Training day) showed that CE (n = 11) and GEE (n = 7) mice made a similar number of head entries and earned a similar number of rewards (**b**) across schedule training (Fs' < 2.0, *ps'* > 0.05). (**c**) GEE (n = 14) mice did not show any difference in locomotion in a novel cage compared to CE (n = 12) (repeated-measures ANOVA (day x treatment group); no interaction: $F_{2,126} = 1.07$, p =

0.35; main effect of day: $F_{2, 126} = 70.05$, $p < 0.0001$; no effect of treatment group: $F_{1,63} = 0.03$, $p = 0.86$). (**d**) However, when examining the locomotion of the same mice as in (c) throughout the trial we observed a trend towards an increase in GEE-induced hyperactivity within the first 5 minutes in the novel cage only on the first day (repeated-measures ANOVA (time x treatment on Day 1); interaction: $F_{19, 1197} = 1.53$, $p = 0.06$; main effect of time: $F_{19, 1197} = 36.70$, $p < 0.0001$; no effect of treatment: $F_{1,63} = 0.51$, $p = 0.48$). During outcome revaluation testing, there were no difference between CE (n = 11) and GEE (n = 7) mice in head-entry behavior (**e**) (2-way ANOVA (Schedule x Day) within Groups) (Fs < 1.20, *ps'* > 0.05) or consumption (**f**) (2-way ANOVA (Outcome x Exposure); interaction: $F < 0.002$, $p > 0.05$; Outcome: $F_{1, 22} = 13.49$, $p < 0.01$). (**g**) A subset of GEE (*n* = 3) and CE (*n* = 3) mice underwent an additional 6 days of RI and RR training after the first outcome revaluation test. A subsequent second outcome revaluation test showed CE mice did not reduce lever-pressing in the devalued state in either context (*ps'* > 0.05), while GEE mice still trended towards a main effect of revaluation state and reduced responding in both RI and RR contexts ($F_{1, 4} = 5.68$, $p = 0.07$) (no interaction). **(h)** An additional cohort of CE (*n* = 6) and GEE (*n* = 6) mice underwent single RI schedule training (no RR schedule training), with CE mice showing similar distribution of lever-presses between Valued and Devalued states, while GEE mice differentially distributed their lever-pressing indicative of goal-directed behavior (one-sample t-tests against 0.5; CE $t = 0.78$, $p > 0.1$; GEE $t = 14.82$, $p < 0.0001$). Error bars equal ± SEM, * = Bonferroni corrected $p < 0.05$.

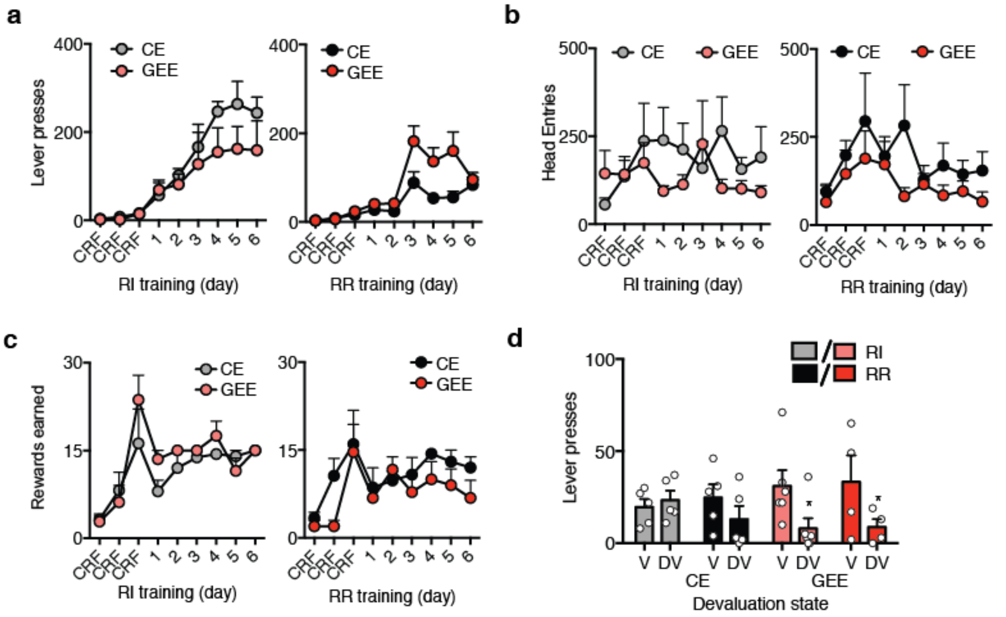

Supplementary Figure 2. Acquisition of goal-directed and habitual actions during in-vivo recording in CE and GEE mice. Following CE or GEE, adult mice were implanted with electrode arrays targeting dorsal striatum. After recovery from surgical procedures, each mouse was trained to press the same lever for the same outcome in two different contexts, with reinforcement controlled by a RI schedule in one context, and an RR schedule in the remaining context. Each day of training, mice were lightly anesthetized prior to the start of the session to plug in the electrode array to the head-stage, which was connected to a MAP (Plexon) system to collect neural activity data. CE (n = 5) and GEE (n = 6) tethered mice learned to lever press. (**a**). A repeated-measures ANOVA (Exposure x Training day) showed that all mice increased the number of lever-presses across training under each schedule (Exposure: Fs' > 7.61, *ps'* < 0.0001). GEE mice made more lever-presses than CE mice only in the RR context (interactions: Fs' > 2.76, *ps'* < 0.05). (**b, c**) However, CE and GEE recording mice did not differ in head entries (Fs' < 0.68, *ps'* > 0.05) or rewards earned (Fs' < 0.67, *ps'* > 0.05) in RI or RR contexts. During an outcome revaluation test (**d**), CE mice showed different patterns of

responding in RI and RR contexts during outcome devaluation testing (repeated-measures ANOVA (Schedule x Revaluation state); interaction: $F_{1,8} = 5.26$, $p = 0.05$), while GEE reduced responding in both RI and RR contexts (main effect of Revaluation state: $F_{1, 8} = 22.25$, $p < 0.01$). Error bars equal ± SEM, * = Bonferroni corrected $p < 0.05$.

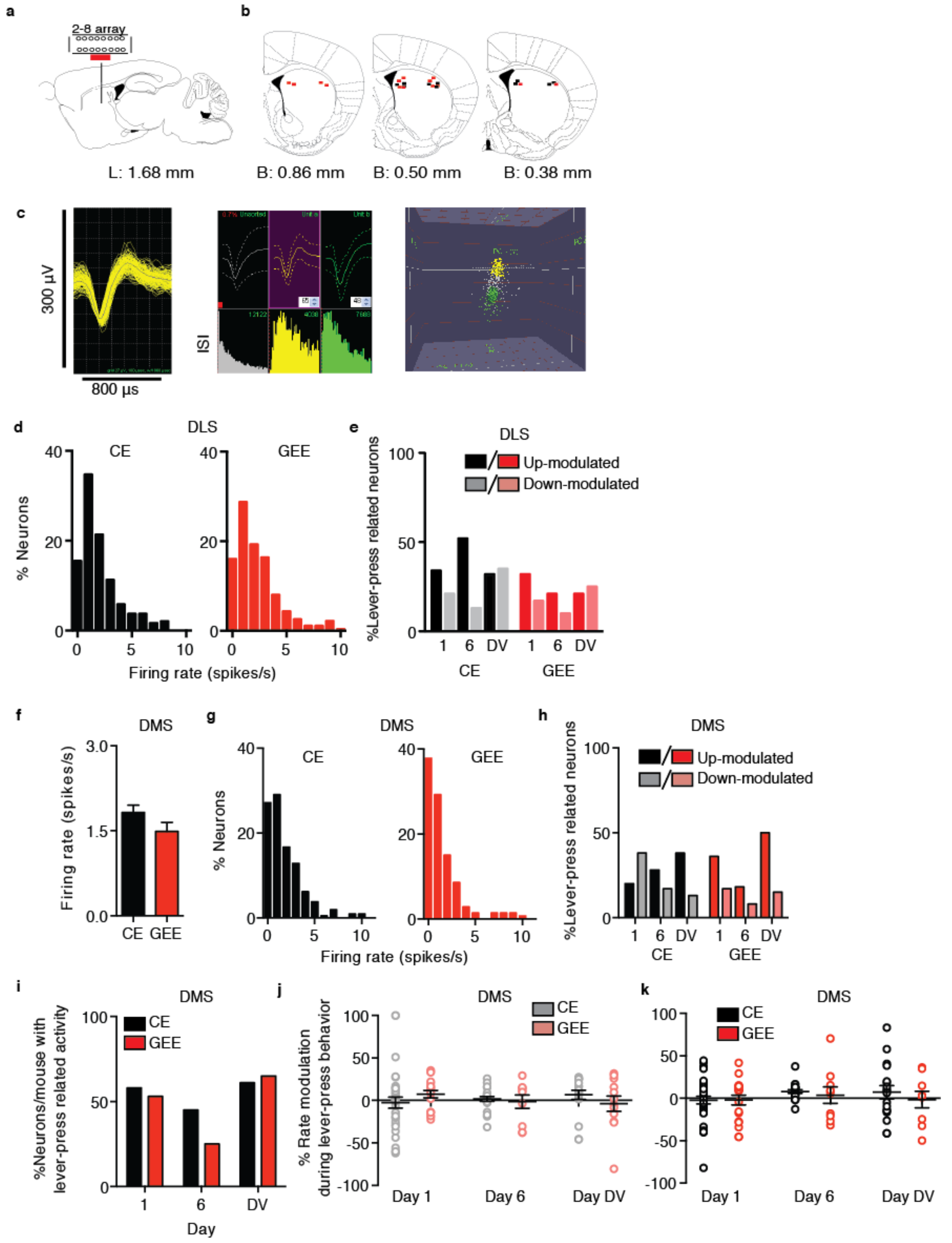

Supplementary Figure 3. Multi-electrode array recordings of DLS and DMS MSNs during acquisition of goal-directed and habitual actions following CE or GEE. (a)

Schematic drawing adapted from Paxinos & Franklin (2004) showing a sagittal mouse section depicting orientation and placement of a 2 x 8 fixed array into dorsal striatum. (**b**) Schematic coronal section drawings adapted from Paxinos & Franklin (2004) showing nissl-stained confirmed placement of electrode arrays into DMS and DLS of CE (black rectangles; *n* = 5) and GEE (red rectangles; *n* =6) mice. (**c**) Putative MSN waveform, all waveforms on that channel, inter-spike interval those waveforms, and cluster of waveforms in PCA space. (**d**) Histograms showing the distribution of baseline firing rates (taken from -5 to -2 sec preceding the lever-press) for all recorded putative DLS MSNs in CE and GEE mice. (**e**) The percentage of recorded DLS neurons per mouse that were up- vs. down-modulated for CE and GEE mice across Day1 and 6 of training, as well as following outcome devaluation (DV). A chi-square analysis performed on each exposure group showed more up- than down-modulated neurons in CE but not GEE mice (CE: $\chi^2 = 5.73$, $p = 0.05$; GEE: $\chi^2 = 0.92$, $p > 0.05$), but there were no significant differences in modulation direction between the groups (*ps* > 0.05). (**f**) The firing rate of putative DMS MSNs did not differ between CE (*n* = 210) and GEE mice (*n* = 140) (two-sided unpaired t-test: $t_{348} = 1.64$, $p > 0.1$), as demonstrated by (**g**) the histograms showing the distribution of baseline firing rates (taken from -5 to -2 sec preceding the lever-press) for all recorded putative DMS MSNs in CE and GEE mice. (**h**) Similar to the DLS, a chi-square analysis performed on each exposure group showed more up- than down-modulated neurons in CE but not GEE mice (CE: $\chi^2 = 6.74$, $p = 0.03$; GEE: $\chi^2 = 4.44$, $p > 0.1$). However, a comparison between CE and GEE groups on each day revealed a difference in modulation direction on the first day of schedule training ($\chi^2 = 3.52$, p <0;05), that was not present on day 6 of schedule training or following outcome devaluation (*ps'* > 0.05).

(**i**) Additionally, a chi-square analysis performed on the %lever-press related DMS neurons per mouse did not reveal a significant difference between CE and GEE mice ($\chi^2 = 4.652$, $p = 0.09$). (**j, k**) Two-sided unpaired t-tests of % rate modulation of a lever-press related DMS neuron's activity during lever-pressing (peak firing rate during lever-press/firing rate during baseline) at each time point examined did not reveal a difference in % rate modulation between CE and GEE mice in either RI (**j**) or RR (**k**) training contexts (ts'< 1.64, *ps'* > 0.1).

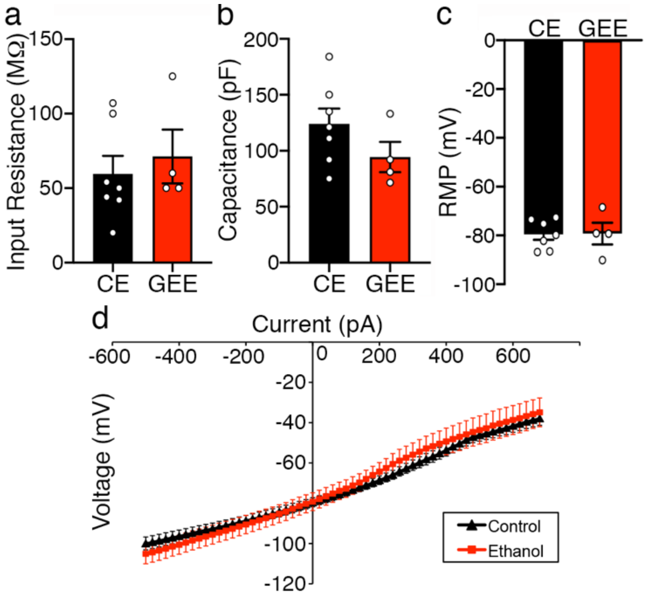

Supplementary Figure 4. Gestational ethanol exposure effects on DLS MSN membrane properties and excitability. Membrane properties and excitability were recorded from CE (n = 7) and GEE (n = 4) DLS MSNs. Input resistance (unpaired t-test, $t_9 = 0.56$, p = 0.59; **a**), whole-cell capacitance (unpaired t-test, $t_9 = 1.40$, p = 0.19; **b**) and resting membrane potential (0 current level; unpaired t-test, $t_9 = 0.06$, p = 0.95; **c**) were not altered after GEE in comparison to CE. (**d**) Plot of current-voltage relationship showing no difference between CE and GEE DLS MSNs (Repeated measures ANOVA (Group x current step) interaction: F59, 531 = 1.06, $p = 0.37$; main effect current step: F59, 531 = 171.5, $p < 0.0001$; main effect Group: F1, 9 = 0.032, $p = 0.86$). Data expressed as mean $\pm$ SEM.

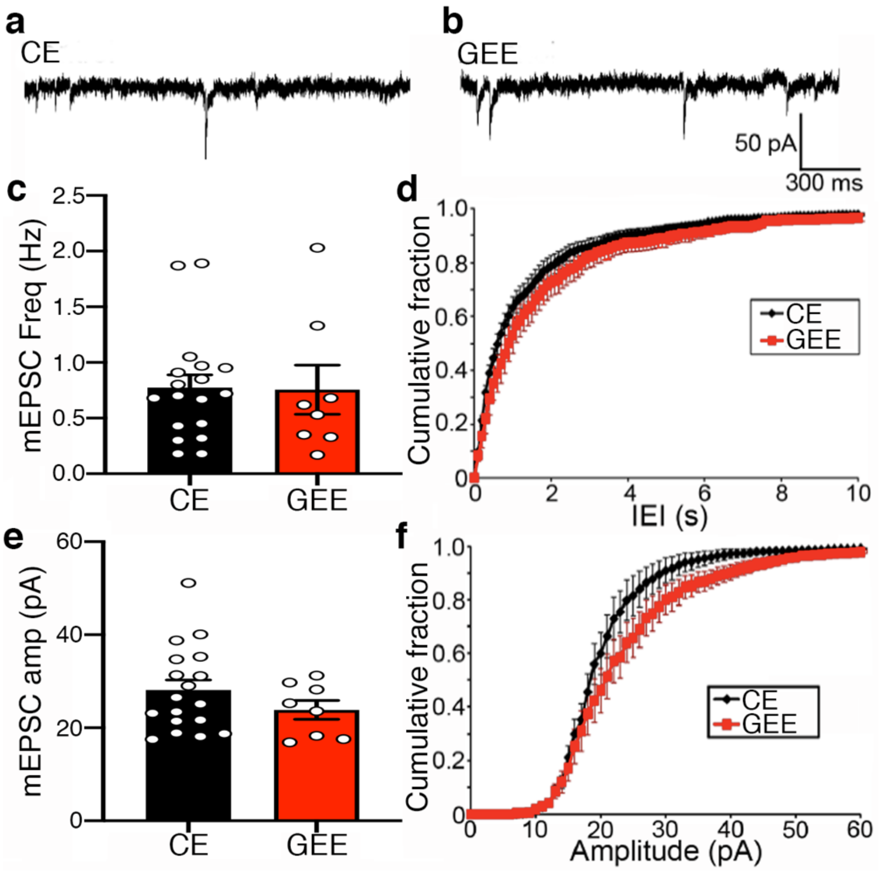

Supplementary Figure 5. Gestational ethanol exposure does not alter miniature excitatory postsynaptic currents (mEPSC) onto MSNs in the DLS. mEPSCs were recorded from CE (n = 18) and GEE (n = 8) DLS MSNs. Representative traces of mEPSCs recorded from CE (**a**) and GEE (**b**) DLS-MSNs. The average (unpaired t-test, $t_{24} = 0.69$, $p = 0.50$; **c**) and cumulative distribution (Kolmogorov-Smirnov test, $p = 0.68$; **d**) for interevent interval did not differ between neurons in the CE and GEE groups. The average (unpaired t-test, $t_{24} = 1.15$, $p = 0.26$; **e**) and cumulative distribution (Kolmogorov-Smirnov test, $p = 0.57$; **f**) of mEPSC amplitudes was also not statistically different between groups. Data expressed as mean $\pm$ SEM.

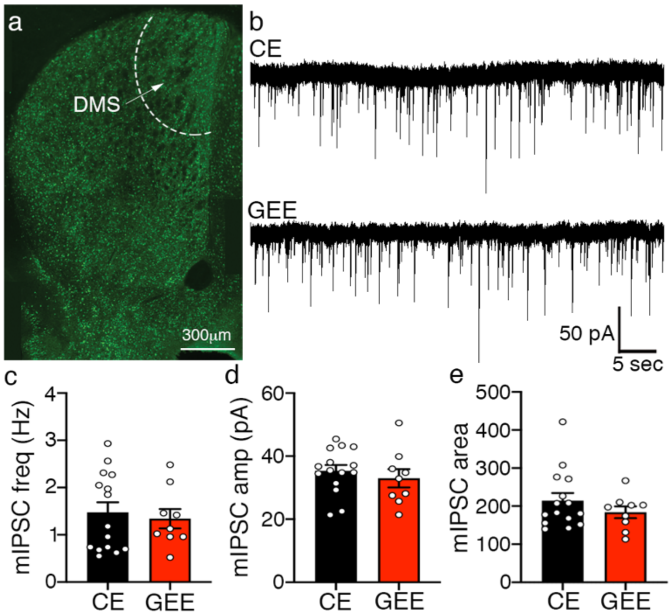

Supplementary Figure 6. GEE does not alter GABAergic transmission onto DMS MSNs. mIPSCs were recorded from CE (n = 15) and GEE (n = 9) DMS MSNs. (**a**) GAD65-GFP slice with the DMS demarcated. (**b**) Representative mIPSCs recorded from CE and GEE DMS-MSNs. (**c-e**) Graphs showing that mIPSC frequency (unpaired t-test, $t_{22} = 0.42$, $p = 0.34$) (**c**), amplitude (unpaired t-test, $t_{22} = 0.73$, $p = 0.24$ (**d**), and area (unpaired t-test, $t_{22} = 1.08$, $p = 0.15$ (**e**) are similar between CE and GEE DMS-MSNs. Error bars equal ± SEM.

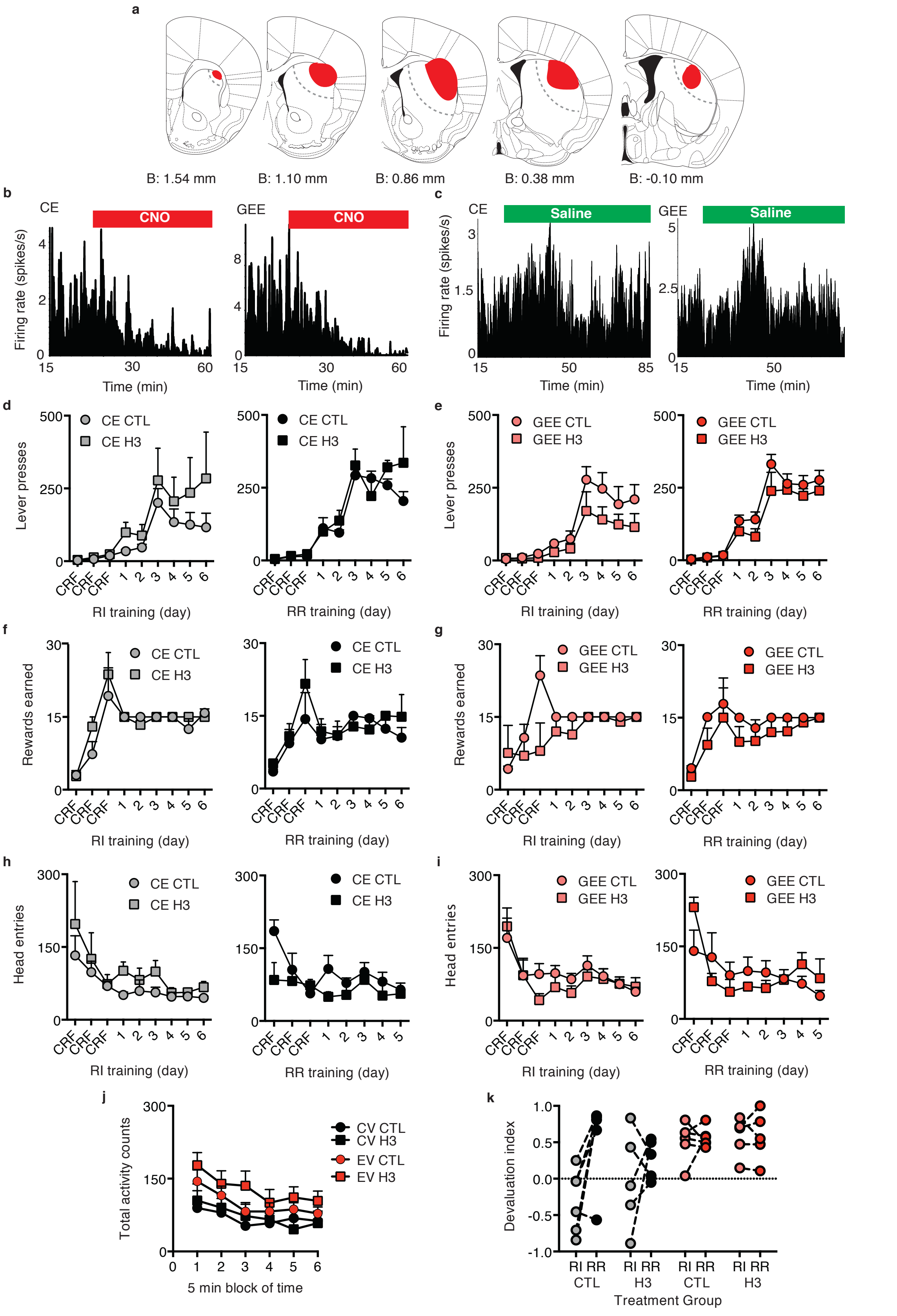
a
B: 1.54 mm
B: 1.10 mm
B: 0.86 mm
B: 0.38 mm
B: -0.10 mm
b
CE
CNO
GEE
CNO
Firing rate (spikes/s)
Time (min)
c
CE
Saline
GEE
Saline
Firing rate (spikes/s)
Time (min)
d
Lever presses
CE CTL
CE H3
RI training (day)
RR training (day)
e
Lever presses
GEE CTL
GEE H3
RI training (day)
RR training (day)
f
Rewards earned
CE CTL
CE H3
RI training (day)
RR training (day)
g
Rewards earned
GEE CTL
GEE H3
RI training (day)
RR training (day)
h
Head entries
CE CTL
CE H3
RI training (day)
RR training (day)
i
Head entries
GEE CTL
GEE H3
RI training (day)
RR training (day)
j
Total activity counts
CV CTL
CV H3
EV CTL
EV H3
5 min block of time
k
Devaluation index
RI RR
CTL
RI RR
H3
RI RR
CTL
RI RR
H3
Treatment Group

Supplementary Figure 7. Effect of CNO activation of $hM3D_q$ receptors on DLS parvalbumin neurons on acquisition of instrumental actions. (**a**) Inclusion area (dotted gray line) and representative spread of $hM3D_q$ expression (red area) through dorsal striatum. (**b, c**) Firing rate of putative MSNs in CE and GEE *Pvalb-cre* mice expressing hM3Dq receptors following systemic injection of CNO and saline.
(**d, e**) All mice increased the number of lever presses across schedule training, with $hM3D_q$ activation not affecting this increase (RI: Fs' > 8.21, *ps'* < 0.0001; RR: Fs' > 27.56, *ps'* < 0.0001. (**f, g**) In general Ctl and $hM3D_q$ mice earned similar rewards across training (main effect of time: Fs' > 5.38, *ps'* < 0.001), with the exception that GEE Ctl ($n = 6$) mice earned more rewards than GEE $hM3D_q$ mice ($n = 5$) on the last day of CRF training in the RI context (i.e. no CNO on board) (Two-way repeated measures (ANOVA (Group x Day) interaction: F8, 80 = 2.87, $p < 0.007$; main effect of time: F8, 80 = 4.49, $p < 0.0001$). (**h, i**) All Ctl and $hM3D_q$ mice decreased head entries similarly across schedule training (main effect of time: Fs' > 3.82, *ps'* < 0.01) (Two-way repeated measures ANOVA (Group x Day). (**j**) During devaluation testing, only CE Ctl mice ($n = 6$) (CE $hM3D_q$ $n = 5$) showed a significant shift in the magnitude of devaluation (two-sided unpaired t-tests: CE Ctl $t_{10} = 3.04$, $p = 0.01$; CE $hM3D_q$ $t_8 = 0.89$, $p > 0.05$; GEE Ctl $t_{10} = 0.33$, $p > 0.05$; GEE $hM3D_q$ $t_8 = 0.05$, $p > 0.05$). (**k**) Examination of locomotor activity following CNO injection did not reveal any significant differences between CE and GEE Ctl and $hM3D_q$ mice (Three-way repeated measures ANOVA) no three-way interaction time x group x treatment), but did reveal a main effect of time (F5, 85 = 15.26, $p < 0.0001$). Error bars equal ± SEM.

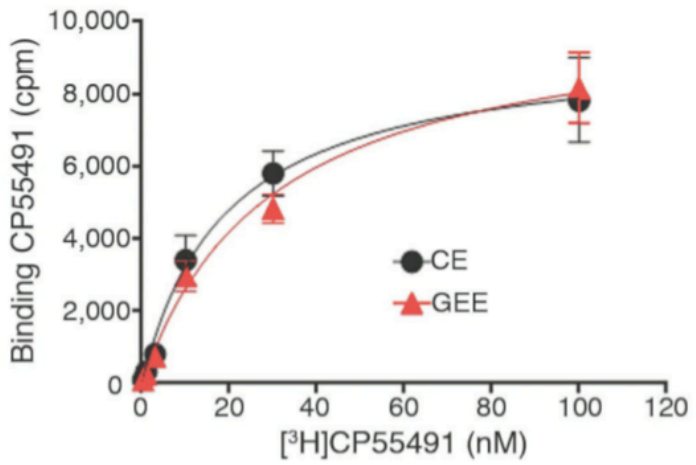

Supplementary Figure 8. Gestational ethanol exposure does not alter CB1 receptor agonist binding in the DLS. Radioligand binding of [^{3}H]-CP55940, an agonist at CB1 and CB2 receptors was used to measure CB1R binding in the DLS of CE (n = 5) and GEE (n = 5) mice. (Bmax: CE 9419 $\pm$ 1100 cpm, GEE 10468 $\pm$ 1068 cpm; unpaired t-test, $t_6 = 0.47$, $p = 0.66$) (Kd: CE 19.07 $\pm$ 6.36 nM, GEE 29.74 $\pm$ 7.52 nM; unpaired t-test, $t_6 = 1.13$, $p = 0.30$). Error bars equal ± SEM.

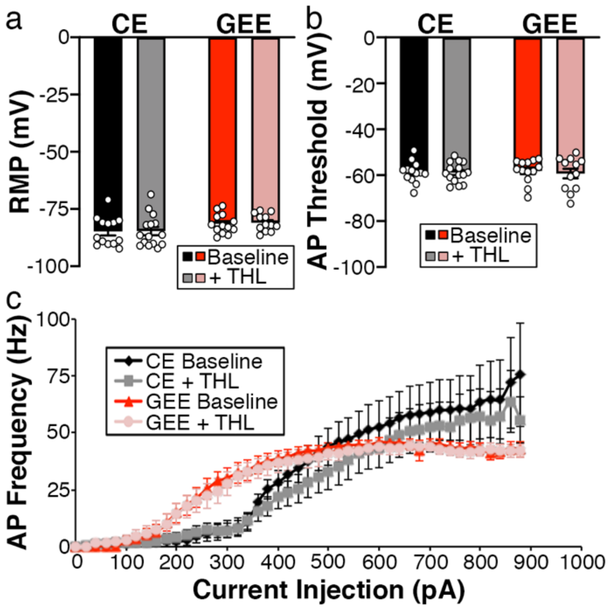

Supplementary Figure 9. Acute application of the DAG-lipase inhibitor THL does not alter resting membrane properties and action potential characteristics. THL was bath applied to CE (n = 13) and GEE (n = 13) MSNs. THL application does not alter (**a**) resting membrane potential (CE baseline: -84.78 $\pm$ 1.76, CE+THL: -84.66 $\pm$ 1.79; unpaired t-test, $t_{26} = 0.05$, p = 0.96; GEE baseline: -81.48 $\pm$ 1.20, GEE+THL: -80.93 $\pm$ 1.08; unpaired t-test, $t_{23} = 0.34$, p = 0.74), (**b**) action potential thresholds (CE baseline: -58.65 $\pm$ 1.29, CE+THL: -58.87 $\pm$ 1.12; unpaired t-test, $t_{26} = 0.13$, p = 0.90; GEE baseline: -58.13 $\pm$ 1.40, GEE+THL: -59.33 $\pm$ 2.09; unpaired t-test, $t_{23} = 0.48$, p = 0.63), or (**c**) current-action potential frequency relationship (CE: 2-way ANOVA (current x drug); interaction: F = 0.06, $p > 0.99$; current: F69, 1820 = 254.8, $p < 0.0001$; GEE: 2-way ANOVA (current x drug); interaction: F = 0.45, $p > 0.99$; current: F69, 1610 = 322.7, $p < 0.0001$) in neurons from either treatment group. Error bars equal ± SEM.

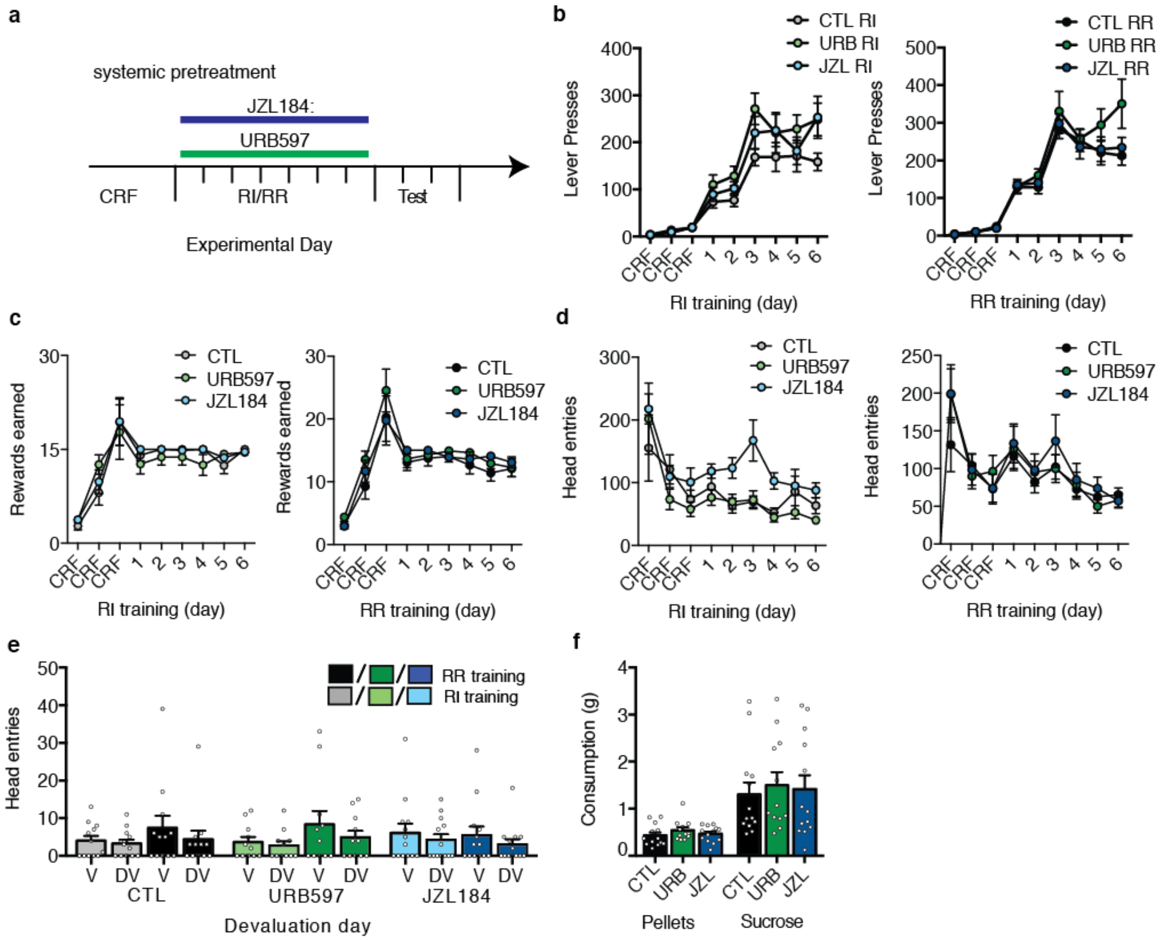

Supplementary Figure 10. Effect of increased endocannabinoid tone on acquisition of goal-directed and habitual actions. Naïve C57Bl/6J mice were pretreated with the FAAH inhibitor URB597 (10 mg/kg, 10 ml/kg) (n = 11) or MAGL inhibitor JZL184 (16 mg/kg, 10 ml/kg) (n = 12), or vehicle (10 ml/kg) (n = 12) 2 hr prior to lever-press training under RI and RR schedules of reinforcement **(a)**. A repeated measures ANOVA (Treatment x Training day) performed for each schedule showed Ctl mice and mice pretreated with URB597 or JZL184 similarly increased the level of lever-pressing **(b)** (interaction: Fs' < 1.5, *ps'* > 0.05; main effect of Training day: Fs' > 72.73, *ps'* < 0.001), earned a similar level of rewards **(c)** (interaction: Fs' < 0.53, *ps'* > 0.05; main effect of Training day: Fs' > 24.15, *ps'* < 0.001), made similar head entries **(d)** (interaction Fs' <

0.68, *ps'* > 0.05; main effect of Training day: Fs' > 11.23, *ps'* < 0.0001), although JZL mice had over all more head entries in the RI context (main effect Treatment: $F_{2, 264} = 5.59$, $p < 0.01$). In the outcome devaluation test **(e)** Only ctl mice reduced head entries in the devalued state (Two-way repeated measures ANOVA) main effect of Revaluation state: $F_{1, 22} = 6.40$, $p < 0.05$) (JZL184 and URB597 mice: Fs' < 3.4, *ps'* > 0.05). During the outcome devaluation test, pretreated and control mice consumed similar amounts of sucrose and pellets **(f)** (Two-way repeated measures ANOVA) interaction (Outcome x Treatment): F < 0.03, $p > 0.05$; main effect of Outcome: $F_{1, 70} = 31.22$, $p < 0.001$). Error bars equal ± SEM, * = Bonferroni corrected $p < 0.05$.

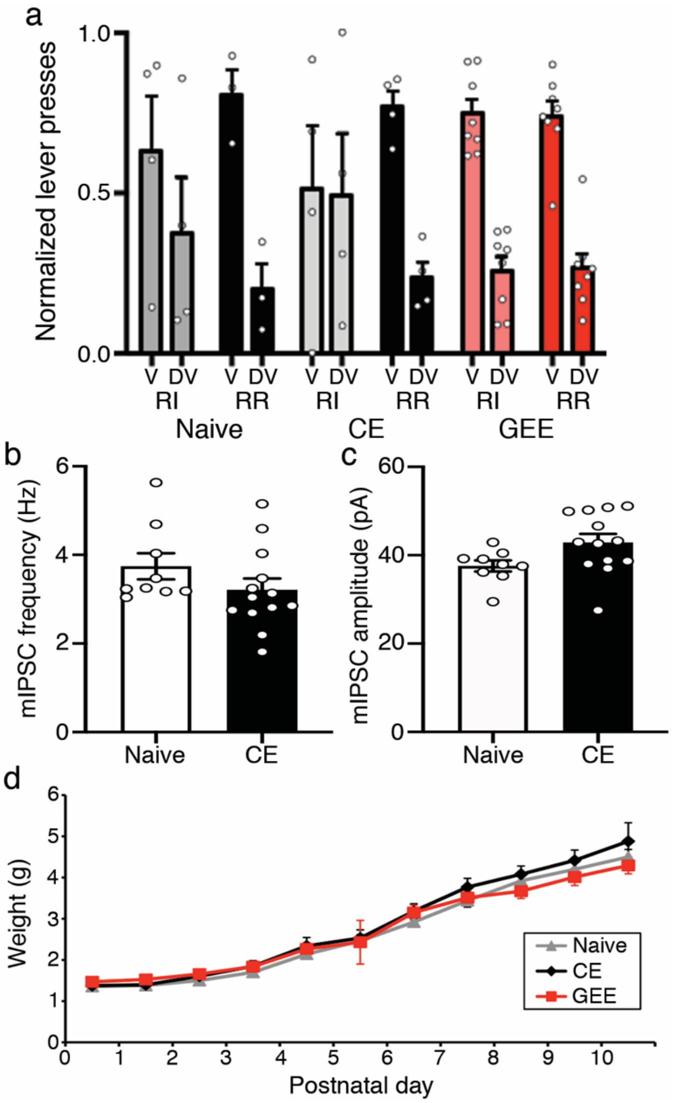

Supplementary Figure 11 Naïve controls. (**a**) Animals that were naïve (n = 4) to the vapor chamber paradigm are not different from CE mice (n = 4) in goal-directed learning and habit formation, with both only reducing lever-pressing in the devalued state in the RR context, while GEE mice (n = 8) reduced in both contexts (two-sided one-sample t-tests: naïve RI $t_3 = 0.73$, $p > 0.10$; naïve RR $t_3 = 3.79$, $p = 0.06$; CE RI $t_3 = 0.06$, $p > 0.10$; CE RR $t_3 = 5.37$, $p < 0.05$; GEE RI $t_7 = 5.62$, $p < 0.001$; GEE RR $t_7 = 5.18$, $p < 0.01$). (**b**) mIPSC frequency recorded in DLS MSNs (naïve: 3.74 $\pm$ 0.29, n = 9; CE: 3.21 $\pm$ 0.26, n= 13; unpaired t-test, $t_{20} = 1.35$, p = 0.19), (**c**) mIPSC amplitude recorded in DLS MSNs

(naïve: 44.36 $\pm$ 2.03, n = 9; CE: 38.34 $\pm$ 2.05, n = 13; unpaired t-test, $t_{20} = 2.07$, p = 0.05), or (**d**) weight during development (repeated-measures ANOVA (postnatal age x treatment group); main effect interaction: F20,230 = 2.36, $p = 0.001$; main effect of postnatal age: F10,230 = 530.3, $p < 0.0001$; no effect of treatment group: F2,23 = 1.07, $p = 0.36$)(Naïve n = 8, CE n = 9, GEE n = 9). Error bars equal ± SEM, * = Bonferroni corrected $p < 0.05$.

SUPPLEMENTARY TABLE

	CE	GEE	repeated measures two-way ANOVA (interaction)
Baseline	41.12 ± 3.06	38.32 ± 2.36	F(1,16) = 0.90, p = 0.36
+ Win	38.08 ± 3.26	37.31 ± 3.43	
Baseline	36.81 ± 2.45	37.42 ± 1.85	F(1,22) = 1.63, p = 0.21
+ AM251	35.40 ± 2.31	39.90 ± 2.57	
Baseline	45.28 ± 2.72	42.10 ± 4.10	F(1,23) = 0.005, p = 0.95
+ THL	42.97 ± 3.69	40.14 ± 1.34	
Baseline	45.57 ± 3.30	41.72 ± 1.42	F(1,17) = 0.24, p = 0.63
+ URB597	42.10 ± 2.61	39.72 ± 1.55	
Baseline	41.73 ± 3.98	41.11 ± 1.48	F(1,19) = 0.13, p = 0.73
+ JZL184	39.60 ± 3.94	39.63 ± 1.81	

Supplementary Table 1. Pharmacological agents that alter the CB system do not affect mIPSC amplitude. The mean amplitude ± SEM of mIPSCs recorded during baseline and plus WIN55,212-2 (1 μM), AM251 (2 μM), THL (10 μM), JZL184 (10 μM), or URB597 (1 μM).
